# Supplementary material for: Metabolic profiling of cancer cells reveals genome-wide crosstalk between transcriptional regulators and metabolism
Source: Nat Commun. 2019 Apr 23;10:1841. doi: 10.1038/s41467-019-09695-9 (PMC6478870; doi:10.1038/s41467-019-09695-9)
Supplement: Supplementary file 1 — Supplementary Information [file 41467_2019_9695_MOESM1_ESM.pdf]

Supplementary Information for

Metabolic profiling of cancer cells reveals genome-wide crosstalk between transcriptional regulators and metabolism

Ortmayr et al.

Supplementary Methods 2
Supplementary Discussion..5
Supplementary Note 11
Supplementary Figures..13
Supplementary References...27

Supplementary Methods

Analysis of tissue signatures across four omics data types

Pair-wise similarity between cell lines was estimated by calculating mutual information[98] between transcriptome[1], proteome[2], drug sensitivity[3], metabolite exchange rates[4] and intracellular metabolome profiles. Euclidian distance was used to estimate similarity in growth rates across cell line pairs. Receiver-operator-characteristic (ROC) curve analysis was then applied to test for similar molecular profiles across cell lines from the same tissue type (main text Figure 1c). To find metabolites featuring significant difference in relative abundances across tissue types we used one-way ANOVA analysis. Statistical significance (i.e. p-values) were corrected for multiple hypothesis testing using the Storey method[5] (i.e. q-values). Metabolites exhibiting a q-value below 0.05 for at least one tissue were considered significantly tissue-dependent.

Measurement of glucose and lactate exchange rates

Uptake of glucose and secretion of lactate was quantified in 54 adherent cell lines from the NCI-60 panel. Supernatants of growing cell cultures were collected every 24 hours for 5 days of cultivation. Samples between 20 and 80% confluence were retained for estimates of glucose uptake and lactate secretion. Residual glucose in culture supernatants was quantified using an enzymatic assay involving coupled glucose oxidase and peroxidase reactions (GOPOD format, K-GLUC, Megazyme, Bray, UK). For each sample, 5 µL supernatant was mixed with 150 µL assay reagent, and incubated at 40°C for one hour before reading absorbance at 510 nm. Lactate was quantified from FIA-TOFMS measurements (as described above) against a standard calibration curve comprising different lactate concentrations in constant RPMI1640 background. To estimate exchange rates, measured amounts of glucose and lactate were fitted with a linear model relating ion intensity to cell numbers over 3 biological replicates. For each cell line estimated curve slopes were multiplied by the corresponding cell line growth rates and normalized by the volume correcting factor (Supplementary Data 1).

RNA interference, siRNA transfection and HIF-1A knockdown

Transfection- and pooled siRNA reagents were obtained from Horizon Discovery Group Company, Lafayette, CO: DharmaFECT 1 siRNA Transfection Reagent (cat. no. T-2001), ON-TARGETplus Human HIF1A siRNA – SMARTpool (cat. no. L-004018-00) and ON-TARGETplus Non-targeting Pool (cat. no. D-001810-10). Upon receipt, siRNAs were resuspended in 1x siRNA buffer (B-002000-UB, diluted 1:5 in sterile-filtered and nuclease-free water) and stored at -20°C. To verify transfection of IGROV1 cells with these reagents, a fluorescent control siRNA (BLOCK-iT Alexa Fluor Red Fluorescent Control, cat. no. 14750100, Thermo Scientific) was transfected to monitor internalization of siRNA into cells using a Nikon Ti-E inverted microscope equipped with an LED illumination system (pE-2, CoolLED, Andover, UK) and a Hamamatsu ORCA (C4742-95-12ER) camera. Cells were counted manually in phase contrast and fluorescence images, respectively, and a transfection efficiency of 92% was calculated (see Supplementary Figure 6). For knockdown and dynamic metabolome profiling experiments, IGROV1 ovarian cancer cells were resuspended in RPMI1640 cell culture medium (5% dFBS, 2 g/L glucose, 2 mM glutamine) without antibiotics, seeded at low cell density (approx. 20% confluence) in 96-well plates and allowed to attach at 37°C and 5% CO_2 for approximately 6 hours. The transfection mix was prepared as per manufacturer's instructions. For each well to be transfected, 0.25 µL transfection reagent were mixed with 9.75 µL serum- and antibiotics-free medium. In parallel, 5 µM stock solutions of siRNA were pre-diluted in a total volume of 10 µL serum- and antibiotics-free medium per well to achieve final concentrations at

transfection of 10, 25 and 50 nM of HIF-1A siRNAs, and 25 nM of the non-targeting control siRNA. After incubating the separate components at room temperature for 5 minutes, both were mixed and incubated for 20 minutes at room temperature. Finally, 20 µL of the medium in each well was replaced by 20 µL transfection mix in three biological replicates per condition. Transfected cell cultures were then incubated at 37°C and 5% CO_2 atmosphere for a total of 144 hours. Nine replicate 96-well plates were prepared: two for metabolomics sampling (using the protocol described in Supplementary Figure 1) at time point: 48, 64, 87 and 111 hours after transfection, and one plate for continuous monitoring of cell confluence in a TECAN Spark 10M plate-reader. Sampling time-points were selected to be between one and two population doublings after transfection (Supplementary Figure 6) to allow for sufficient reduction of HIF-1A protein levels by dilution during growth. The presence of fluorescent control siRNA in separate control cells was verified as described above at all sampling time-points to ensure sustained transfection. Cell extracts were analyzed by flow-injection TOFMS as described above. Analysis of time-dependent fold-changes relative to the non-targeting control was performed as described in detail in ref. [6].

Associations between TR activity and drug action

In this analysis we used publicly available data (http://dtp.cancer.gov/mtargets/mt_index.html) on the sensitivity of the NCI-60 cell lines to 21738 compounds. Cell-line drug sensitivity is reported as the normalized Z-score of the GI_{50} values (e.g. the concentration of a compound that causes 50% growth inhibition, relative to the no drug control). Here we considered only the 130 FDA approved drugs (Supplementary Data 4). Some of the FDA-approved anticancer drugs were tested in the NCI-60 screen twice, resulting in the overall selection of 187 drug sensitivity profiles across the NCI-60 tumor cell panel. The influence of TRs on drug susceptibility is inferred using a multivariate statistical analysis to decouple the contribution of different tissues of origin from changes in TR activity. For each TR i and drug j, drug susceptibility (S) is modeled as a linear combination of changes in TR activity (TR^{α}) and tissue-specific offsets values (β):

$$S_{\mathrm{TR_i}}^{\mathrm{d_j}} = \lambda \cdot \mathrm{TR_i^{\alpha}} + \beta_{\mathrm{Lung}} + \beta_{\mathrm{Colon}} + \beta_{\mathrm{Melanoma}} + \beta_{\mathrm{Prostate}} + \beta_{\mathrm{Kidney}} + \beta_{\mathrm{CNS}} + \beta_{\mathrm{Breast}} \qquad \text{Supplementary eq. (1)}$$

λ and β values are estimated using least squares fitting. The significance of the association between changes in TR activity and drug susceptibility is estimated by calculating the p-value of the Spearman correlation between TR activity and drug susceptibility after removal of tissue-specific offset β values across the 54 cell lines (i.e. $S_{\mathrm{TR_i}}^{\mathrm{d_j}} - \beta_{\mathrm{Lung}} - \ldots - \beta_{\mathrm{Breast}}$). The inferred slope λ, p-value significance and Z-score normalized β_{Kidney} values are used in Figure 3e-f, to visualize the inferred relationships between drug susceptibility and changes in VHL and HIF-1 activity. In order to find significant associations between TRs with common biological functions and drug modes of action (Figure 3d), we first use The Database for Annotation, Visualization and Integrated Discovery (DAVID - https://david.ncifcrf.gov/) to identify 131 clusters of functionally related TRs based on associated GO-terms (Supplementary Data 3). Subsequently we used a hypergeometric test to analyze significant TR-drug associations (i.e. p-value ≤ 2.67e-04, Bonferroni-adjusted threshold) and look for an enrichment of TR-functional clusters across different drug MoAs (Supplementary Data 4).

Prediction of metabolite-TR effectors

Because of the poor correlation between protein levels and NCA estimated TR activities (Supplementary Figure 5), we systematically investigate whether variation in TR activities across cell lines could be explained by alternative models. Instead of assuming a base model where the TR activity is a simple linear function of protein abundance, we model TR activity as a function of TR protein levels and the post-translational regulatory functions of metabolites and/or kinases, individually or in combination as follows:

$$\mathrm{TR}^{\mathrm{j}}_{\mathrm{activity}} = \frac{K_1}{1+\frac{M_i}{K_2}} \cdot P_{\mathrm{TR}^{\mathrm{j}}} + K_3$$ for a metabolite inhibitor, Supplementary Eq. (2)

$$\mathrm{TR}^{\mathrm{j}}_{\mathrm{activity}} = \frac{K_1 \cdot M_{\mathrm{i}}}{M_{\mathrm{i}}+K_2} \cdot P_{\mathrm{TR}^{\mathrm{j}}} + K_3$$ for a metabolite activator, Supplementary Eq. (3)

$$\mathrm{TR}^{\mathrm{j}}_{\mathrm{activity}} = \frac{K_1}{1+\frac{P_{\mathrm{x}}}{K_2}} \cdot P_{\mathrm{TR}^{\mathrm{j}}} + K_3$$ for a kinase inhibitor, Supplementary Eq. (4)

$$\mathrm{TR}^{\mathrm{j}}_{\mathrm{activity}} = \frac{K_1 \cdot P_{\mathrm{x}}}{P_{\mathrm{x}}+K_2} \cdot P_{\mathrm{TR}^{\mathrm{j}}} + K_3$$ for a kinase activator, Supplementary Eq. (5)

$$\mathrm{TR}^{\mathrm{j}}_{\mathrm{activity}} = \left(\frac{K_1 \cdot P_{\mathrm{x}}}{P_{\mathrm{x}}+K_2} + \frac{K_1}{1+\frac{M_{\mathrm{i}}}{K_4}}\right) \cdot P_{\mathrm{TR}^{\mathrm{j}}} + K_3$$ for a kinase activator & metabolite inhibitor, Suppl. Eq. (6)

$$\mathrm{TR}^{\mathrm{j}}_{\mathrm{activity}} = \left(\frac{K_1}{1+\frac{P_{\mathrm{x}}}{K_2}} + \frac{K_1}{1+\frac{M_{\mathrm{i}}}{K_4}}\right) \cdot P_{\mathrm{TR}^{\mathrm{j}}} + K_3$$ for a kinase inhibitor & metabolite inhibitor, Suppl. Eq. (7)

$$\mathrm{TR}^{\mathrm{j}}_{\mathrm{activity}} = \left(\frac{K_1 \cdot P_{\mathrm{x}}}{P_{\mathrm{x}}+K_2} + \frac{K_1 \cdot M_{\mathrm{i}}}{M_{\mathrm{i}}+K_4}\right) \cdot P_{\mathrm{TR}^{\mathrm{j}}} + K_3$$ for a kinase activator & metabolite activator, Suppl. Eq.(8)

$$\mathrm{TR}^{\mathrm{j}}_{\mathrm{activity}} = \left(\frac{K_1}{1+\frac{P_{\mathrm{x}}}{K_2}} + \frac{K_1 \cdot M_i}{M_{\mathrm{i}}+K_4}\right) \cdot P_{\mathrm{TR}^{\mathrm{j}}} + K_3$$ for a kinase inhibitor & metabolite activator. Suppl. Eq. (9)

Where $K_{1,2,3,4}$ are free parameters in the model, $P^{\mathrm{j}}_{\mathrm{TR}}$is the relative protein abundance associated to TR^{j}, P_{x} represents the relative protein abundance of kinase x and M_{i} the level of metabolite i.

For models in which we assume the non/inhibitory actions of a single metabolite or kinase, we use a non-linear model fitting scheme to find the best set of 3 parameters to describe changes in TR activity across cell lines. To minimize the probability of local minima, we adopted the GlobalSearch function of Matlab and 50,000 starting points. By using the same approach, we tested all possible combinations between pairs of metabolites and kinases that can act either as activators and/or inhibitors, to find the best set of 4 parameters that describe TR activity. For each pair or triplet of TRs and metabolites and/or kinases we estimate the Mean Squared Error (MSE) associated to each tested model. In addition, we estimated the MSE when fixing the model parameters and randomly permuting TR activity, TR protein, metabolite and kinase levels across cell lines ($\mathrm{MSE}^{\mathrm{e}}$). For each TR we then repeat model fitting, each time by randomly shuffling metabolite and kinases levels. We used this approach to empirically assess the significance of each model in better explaining TR activity. To this end, we calculated MSE values for each model in which kinases and metabolite levels were randomly permuted ($\mathrm{MSE}_{\mathrm{Random}}$ and $\mathrm{MSE}^{\mathrm{e}}_{\mathrm{Random}}$). Finally we retain only those models in which MSE and $\mathrm{MSE}^{\mathrm{e}}$ are both above the 0.1% of the respective distributions of $\mathrm{MSE}_{\mathrm{Random}}$ and $\mathrm{MSE}^{\mathrm{e}}_{\mathrm{Random}}$ (Supplementary Data 5). The proteome dataset (ArrayExpress, project accession: E-PROT-2) reports protein levels for 100 annotated TRs and 63 kinases, and to reduce computation time we here consider only differentially abundant metabolites annotated to KEGG identifiers (260 metabolites selected). Fitting analysis was performed in Matlab using the "fitnlm" function, on a cluster with 900 computational nodes.

Supplementary Discussion

Mathematical modeling of metabolic pathways and TR-metabolite correlations

Here, we describe a set of assumptions sufficient to formally explain and describe the existence of a dependency between metabolite abundance and TR activity. We assume that a TR has the ability to directly regulate the flux demand or supply of a linear metabolic pathway, which consists of 4 intermediate metabolites, 3 irreversible and 1 reversible reactions, which we assume to follow Michaelis-Menten kinetics. A schematic representation is illustrated in Supplementary Figure 5, panel a.

TRs can regulate reaction rates by changing the abundance of enzymes. In reactions that operate above maximum enzyme capacity regulation of enzyme abundance confers to the TR high control on pathway usage. In such a case, the flux through the reaction is largely proportional to the amount of enzyme (E_i), which we assume to be a monotonous function of TR activity. Specifically, we assume enzyme abundance to be a positive linear function of TR activity. The individual control coefficient that is defined as the slope of the log–log plot of flux versus TR activity reflects the influence of TR on the flux through the pathway[7]. In this particular example where the flux decreases by the same relative amount as the TR activity, the value of the control coefficient is 1. Hence, at steady state, flux through the pathway is set by and proportional to TR activity.

$V_{input} = K_{cat,i} * E_i$, where $E_i \propto \beta * TR_{activity}$ Supplementary Equation (10)

Notably, functional regulations where TRs are capable of regulating pathway activity are the associations we aim to extrapolate from our combined analysis of the transcriptome and metabolome of cancer cell lines. To this end, if we assume that the intermediate reactions in the pathways operate well below maximum flux capacity we can derive direct functional dependencies between flux, TR activity and substrates levels that holds true for irreversible reactions:

$$V_1 = V_{\max_1} \cdot \frac{M_1}{M_1 + K_{m_1}}$$ Supplementary Equation (11)

$$M_1 = \frac{K_{m_1} \cdot v_{input}}{V_{\max_1} - v_{input}}$$ Supplementary Equation (12)

If $v_{input} \ll V_{\max_1}$:

$$M_1 = \frac{K_{m_1}}{V_{\max_1}} \cdot v_{input} = \frac{K_{m_1}}{V_{\max_1}} \cdot K_{cat_1} \cdot \beta \cdot \mathrm{TR}_{activity}$$ Supplementary Equation (13)

as well as for reversible reactions:

$$V_2 = \frac{V_{\max_2} \cdot \left(M_2 - \frac{M_3}{K_{eq}}\right)}{K_{m_2,M_2} \cdot \left(1 - \frac{M_3}{K_{m_2,M_3}}\right) + M_2}$$ Supplementary Equation (14)

$$V_3 = V_{\max_3} \left(\frac{M_3}{K_{m_3,M_3} + M_3}\right)$$ Supplementary Equation (15)

$$M_2 = \frac{v_{input} \cdot K_{m_2,M_2}}{V_{\max_2} - v_{input}} + \frac{v_{input} \cdot K_{m_3,M_3} \cdot V_{\max_2}}{K_{eq}(V_{\max_2} - v_{input})(V_{\max_3} - v_{input})} + \frac{v_{input}^2 \cdot K_{m_2,M_3}}{(V_{\max_2} - v_{input})(V_{\max_3} - v_{input})}$$ Supplementary Eq. (16)

$$M_2 = v_{input} \cdot \left(\frac{K_{m_2,M_2}}{V_{\max_2}} + \frac{K_{m_3,M_3}}{K_{eq} \cdot V_{\max_3}}\right) = \beta \cdot \mathrm{TR}_{activity} \cdot \left(\frac{K_{m_2,M_2}}{V_{\max_2}} + \frac{K_{m_3,M_3}}{K_{eq} \cdot V_{\max_3}}\right)$$ Supplementary Eq. (17)

where M represents metabolite abundance, v_{input} is the flux through the pathway at steady-state, K_m, K_{cat} and V_{max} are the Michaelis-Menten constant, the catalyst rate constant and maximum flux capacity, respectively. Notably, kinetic parameters (i.e. K_{cat} and V_{max}) can be assumed to be largely conserved across different cell lines, and enzyme levels of reactions mainly regulated by substrate abundance are expected to exhibit little fluctuations. In support of this assumption, we observed that the median fluctuation of protein levels, estimated as the standard deviation of protein levels across cell lines divided by the corresponding average values (i.e. coefficient of variance), is below 50%. Hence, for pathways in which steady-state flux is directly regulated by activity of one TR, and intermediate reactions are governed by substrate abundances, one could expect to find a correlative signature between the relative TR activity and metabolite abundances across cell lines, similarly to the simplified metabolic toy model described here.

It is worth noting that the incidence of metabolic reactions that operate above or below maximum enzyme capacity is still a matter of debate. While recent experimental evidence demonstrates that nearly 70% of reactions in central carbon metabolism are likely to operate at enzyme saturation[8] (i.e. substrate abundances exceeding reactions K_m), a growing body of evidence advocates for a scenario where a large array of post-translation inhibitory mechanisms can increase the apparent K_m of metabolic reactions[9,10]. This possibility might shift the balance in favor of metabolic reactions to be mainly governed by substrate abundances, supporting the large relevance that TR-metabolite associations might have in identifying functional regulation of TRs on cell metabolism.

TR regulatory activity in metabolic pathways – example associations

Tumor suppressor TP53. p53, one of the most well-studied tumor suppressor proteins[11,12], significantly correlates with levels of intermediates in nucleotide metabolism (q-value < 0.05), such as purine biosynthesis and nucleotide sugar metabolism and arginine/proline metabolism. Both associations are consistent with previous experimental evidence[5–7] directly pointing to the well-known functional role of p53 in allocating metabolic resources for DNA repair, DNA replication and cell division[11–13]and regulation of arginine and proline metabolism[14] (see also next section).

Proline metabolism. In recent years, proline metabolism has received considerable attention as a source of NAD/NADP regeneration[15], but also as a central integration point of signaling information[16]. While to date, only few cancer-related TRs have been associated to the regulation of proline metabolism, we here found TR-proline metabolism associations recapitulating known regulatory interactions (KEGG pathway enrichment q-value < 0.05), such as the induction of proline catabolism by tumor suppressor p53[14] (Figure 3a), as well as the regulation of proline metabolism by ATF4 as part of the amino acid starvation response pathway[17,18]. Overall, we found 197 TRs that can potentially impinge on proline metabolism (q-value < 0.05), such as EGR1[19,20] and EGR2[21], REL-family transcriptional regulators[22] and ATF-family TRs[23] (full list in Supplementary Data 3).

MYC and its interactome. MYC is a global regulator that takes part in a number of growth-promoting signaling pathways, many of which ultimately impinge on metabolism[24]. Discovered many years ago and recognized for its frequent hyper-activation in human cancers, MYC is among the most well-studied oncogenes[25,26]. MYC forms stable dimers with the MAX protein to gain specific DNA-binding activity[27]. The formation of MYC/MAX dimers is affected by MAX-binding proteins, most prominently from the MXD family of MAX dimerization proteins[28,29]. MXD proteins are transcriptional repressors that act as MYC antagonists not only by competing for available MAX with MYC, but MAX/MXD and MYC/MAX dimers also compete for DNA binding sites. MXD proteins and other members of the dense network of TRs around MYC (the so-called MYC interactome) can hence attenuate

MYC-dependent gene expression by a plethora of homo- and hetero-dimerization events[28,29], and affect the functional outcome of MYC activation/inhibition[30].

Consistent with the current working hypothesis of a MYC interactome, from which MYC activity emerges as a complex function of protein-protein interactions, we found that, while MYC itself exhibits rather weak associations with metabolic pathways (Supplementary Figure 6, panel b), metabolites associated with MAX and other members of the MYC interactome (MXD1, MXD4, MLX, MXI1) were overrepresented in several central metabolic pathways (Supplementary Figure 6, panel b). It is worth noting that the evidence provided here, based on direct measurements of metabolic intermediates and their associations with TR activity, is complementary to previous reports that were predominantly based on differential gene expression and knock-down experiments.

Nuclear factor kappa B (NF-κB). Nuclear factor kappa B (NF-κB) complexes act as transcriptional regulators of general stress responses[22,31], inflammatory responses and cellular homeostasis with widespread implications for cancerogenesis[32]. The role of NF-κB dimers extends into metabolism, where NF-κB has been associated with p53-mediated metabolic reprogramming of energy metabolism[33,34]. NF-κB complexes can be homo- or heterodimers of different subunits, including NF-κB1/p105, NF-κB2/p10, RelA/p65 and RelB, which exhibit different specificity for DNA elements[35,36] and can hence trigger transcription of specific sets of target genes. NF-κB activity is regulated by members of the inhibitor of κB (IκB) protein family that can affect NF-κB localization[37] by masking its nuclear localization signal sequence. Our metabolome-based TR-metabolite associations hint at metabolic functions specific of different NF-κB complexes (Supplementary Figure 6, panel c). In glycolysis and pentose phosphate pathway, we found strong associations of NF-κB2 and RelA. Similarly, Bcl-3, a member of the IκB protein family, exhibited a strong association with pentose phosphate pathway (Figure 2 in the main text). These associations are consistent with the known gene targets of NF-κB1 and RelA (Figure 2 in the main text), while interactions of NF-κB2, RelB and Bcl-3 with central carbon metabolism were not annotated in the TR-gene interaction network.

Nuclear receptor Rev-ErbA-Alpha (NR1D1). The NR1D1 gene encodes the heme-responsive nuclear receptor Rev-ErbAα, a sequence-specific transcription factor[38]. By acting predominantly as a transcriptional repressor, Rev-ErbA regulates a number of cellular processes, including lipid metabolism and the circadian rhythm[39,40]. In cancer, Rev-ErbA is often found co-expressed with ERBB2[41], encoding the Her2 oncogene, and there has been considerable interest in targeting Rev-ErbA and the frequent disruption of the circadian clock in cancer for therapeutical purposes[42,43].

Among metabolites associated with Rev-ErbA-Alpha in our TR-metabolite association network, we find associations to lipid metabolism and membrane biogenesis (glycerophospholipid metabolism, choline metabolism), as well as to one-carbon-, carbohydrate-, purine- and amino acid metabolism (histidine, glutamine and glutamate). A similar association to lipid metabolism has been previously suggested based on changes in gene expression and altered lipid metabolism phenotypes in gene knock-down experiments[40].

Mapping oncogenic TRs to *in vivo* metabolic reprogramming – renal, lung and colon cancer

In the following sections, we discuss our prediction of TR drivers responsible for metabolic rearrangements in cohorts of patients with renal, lung or colon cancer. The predictions are based on a novel TR-metabolite association network established *in vitro*. The interplay between transcriptional regulation and metabolism is investigated by direct experimental measurements of intracellular metabolite levels and changes in TR activity across 53 cell lines from the NCI60 panel of human tumor cell lines (see main text and Methods section for full detail). Our integrative analysis of TR-metabolite associations and *in vivo* metabolic changes between cancer-

and healthy tissue can help to identify transcriptional regulators that directly contribute to the metabolic rearrangement and aid the therapeutically relevant subtyping of cancers and patients.

Clear-cell renal cell carcinoma. Clear-cell renal cell carcinoma (ccRCC) constitutes more than 70% of all renal cancers[44]. Its genetic basis has been extensively characterized[45–47], and paints a picture in which metabolism plays a crucial role in the development and manifestation of ccRCC[48,49]. Hakimi et al. reported the metabolic profiles of matched ccRCC/normal tissue pairs obtained from 138 patients[49]. After excluding metabolites with more than 10 missing values, we matched 134 metabolites to our metabolomics dataset, of which 77 were found and quantified in the patient samples. Supplementary Figure 7 (panel a) summarizes the metabolites and the corresponding strength of associations with the top 1% most significant transcriptional regulators in our analysis (see Methods section and Figure 3 in the main text). HIF-1α shows the strongest associations to the largest number of metabolites found in patient samples (Supplementary Figure 7, panel a). A ranking of the top 15 predictor metabolites (evaluated by the product of association strength and absolute metabolite Fold-Change) for HIF-1α and the other top 1% predicted TRs is provided in Supplementary Data Table 3.

Lung cancer. Lung cancers are most frequently associated with mutations in TP53 (46% of cases)[50], a type of oncogenic mutation that is shared with a wide range of cancer types.
In the cohort of matched lung cancer/normal fibroblast cells reported by Chaudhri et al.[51], we matched 209 metabolites to our TR-metabolite association network, of which 80 were detected and quantified in the lung cancer cells, with mean absolute fold changes ranging from 1.005 (creatine) to 2.9 (phosphoenolpyruvate). A summary of the metabolites and the corresponding strength of association with the top 1% of TRs recapitulating the observed changes between lung cancer and normal fibroblasts in the dataset is given in Supplementary Figure 7, panel b. The median association strength to this set of metabolites was highest for NFYB, followed by YBX1, NFYC, GFI1, FOXN1, HOXC13 and AATR. Several metabolites exhibiting a high absolute fold change showed a consistently strong association to the seven TRs. This group of metabolites mainly included long-chain fatty acids such as arachidonate, eicosapentaenoate, adrenate and docosapentaenoate. A list of the top 15 predictive metabolites for each TR is provided in Supplementary Data Table 3. The fact that p53, despite being among the most frequently mutated genes in lung cancer[50], is not among the top-ranking TRs likely indicates that there are downstream TRs which can more directly explain the observed metabolic reprogramming between lung cancer cells and normal fibroblasts. Notably, several of the top 1% most significant TRs found in our analysis are linked to p53, including GFI1[52,53] and AATF[54].
The top-ranked TRs NFYB and NFYC are two subunits of the heterotrimeric NF-Y complex, a regulator of cell cycle genes and proliferation[55]. NF-Y appears to act as a downstream effector of both wild-type[56] and mutant p53[57], interestingly changing its role from transcriptional repressor to a transcriptional activator in the latter case. Recently, NF-Y has been described in context of alterations in metabolism[58], including lipid and fatty acid metabolism, glycolysis as well as TCA cycle, where NF-Y binding sites are particularly frequent[58]. Several of the predicted TRs have been associated with lung cancers, including YBX1 (YB-1)[59], GFI1[60] and HOXC13[61].

Colon cancer. Human colorectal cancers are frequently associated with mutations in APC, TP53 and SMAD4, implying among others an involvement of the Wnt signaling pathway[62,63]. Advanced and aggressive colon cancers frequently show enhanced chromosome instability, mutations in p53 and defective DNA damage response[64].
Hu et al. compared the concentrations of five TCA cycle intermediates in a cohort of 10 patients with colon cancer, analyzing 10 matched pairs of cancer/normal tissue samples[65]. The five reported metabolites exhibited mean absolute fold changes ranging from 1.2 (pyruvate) to 6.6 (citrate) (Supplementary Figure 7, panel c). Among the top 7 predicted TRs (Figure 3 in the main text) that explain the observed metabolic rearrangements in the colon cancer cohort, RELB showed the strongest association to the five metabolites, followed by CBX7,

ZNF217, MLLT10 and HNF4G (Supplementary Figure 7, panel c). CEBPE and POU2F2 showed minor associations to only 2 metabolites each. Clearly, given the small number of metabolites quantified in the colon cancer samples, our prediction of an involvement of these TRs would have to be substantiated with additional data (i.e. more quantified metabolites).
Here, we predict an involvement of RELB, a subunit of the NF-κB complex that is involved in inflammation and plays a critical role in malignant transformation and cancer progression[66], with particular relevance in colitis-associated cancers[67,68]. Notably, several of the predicted TRs have previously been associated with signaling pathways that are de-regulated in colon cancer (e.g. Wnt[62,63] and SMAD[69] signaling), including CBX7[70,71], MLLT10[72] and ZNF217[73,74].

Metabolite and/or kinase effectors of TR activity – example predictions

In the following sections, we provide selected examples for regulatory interactions affecting TR activity, as predicted *in silico* using non-linear model analysis to integrate cellular information across three layers, i.e. metabolome, proteome and transcriptome (see main text and Methods section).

Choline. Among the most highly connected metabolites predicted by our model-based fitting analysis is choline (118 interactions with 36 TRs), substrate of the first step in the biosynthesis of phosphatidylcholine, a key component of eukaryotic cellular membranes. Recent studies have revealed a deep interplay between oncogenic signaling and choline metabolism, such that altered choline levels have become a metabolic hallmark of malignant transformation[75]. Our results support this key role, suggesting that choline availability could trigger pleiotropic transcriptional changes.

EIF2AK2/PKR activation by oxidative stress. Eukaryotic Translation Initiation Factor 2 Alpha Kinase 2 (EIF2AK2, also known as PKR) is a regulatory protein acting on eukaryotic initiation factors (eIFs) that mediate the initiation of protein synthesis[76]. In response to various stress signals[77] such as oxidative stress[78,79], EIF2AK2/PKR phosphorylates the subunit α of eIF2. This prevents the assembly of the translation initiation complex, which effectively inhibits mRNA translation[76]. Furthermore, EIF2AK2 acts as part of a signaling pathway that can activate NF-κB[80,81], an important stress response mediator, and inflammation[82].
Here, we predict an interaction between EIF2AK2 and oxidized glutathione (GSSG), which is part of the intracellular redox buffer GSH/GSSG, in combination with several different kinases (Supplementary Data 5). As cells face oxidative stress, excess reactive oxygen species (ROS) are neutralized in part through oxidation of reduced glutathione (GSH), leading to the accumulation of GSSG while the GSH pool is depleted. Serving as a signal for oxidative stress, the predicted interaction may hence represent a mechanism underlying the oxidative stress-mediated activation of EIF2AK2/PKR. The only other interactions predicted for EIF2AK2 are with choline and N-acetylserotonin, in combination with CKB and CKM kinase, respectively.

Effectors of tumor suppressor/oncogene p53. The extensively studied tumor suppressor p53[11,83] has been associated with a plethora of cellular processes, from apoptosis induction[84] and the DNA damage response[85,86] to metabolism[87,88]. Here, our model fitting-based analysis indicates an influence of ATP and fatty acid metabolites such as lipoic acid and myristic acid, as well as the polyamine intermediate N-acetylspermine and the cholesterol metabolite hydroxycholesterol on p53 activity (Supplementary Data 5).
ATP was previously found to directly bind and stabilize p53-DNA complexes[89]. Interestingly, the interaction with p53 was one of only two interactions we predicted for ATP, the second one being with interleukin enhancer-binding factor 2 (Ilf2). Lipoic acid has been attributed a potential involvement in preventing p53 protein degradation[90], while myristic acid, as a representative of saturated fatty acids, can act as a negative

regulator of the DNA damage response[91] to promote cell proliferation. N-acetylspermine is an intermediate in the conversion of spermine to putrescine, catalyzed by spermidine/spermine N(1)-acetyltransferase (SAT1). The polyamines spermine, spermidine and putrescine are required for cell growth[92], and have been suggested to engage in regulatory interplay with p53[93,94]. Hydroxycholesterol is an oxygenation product of cholesterol[95], which has been suggested to act on p53 activity through modulation of the estrogen receptor in breast cancer[96].

Taken together, we predict several small-molecule compounds that could potentially affect p53 activity. Given the drastically different roles of p53 in a wild-type versus mutant p53 setting[97], the therapeutic relevance of these interactions remains to be elucidated. Searching for drug molecules chemically similar to predicted effectors of p53, we found palm oil to potentially interfere with p53 activity, an effect already suggested in the literature[98,99].

Regulation of diverse cellular processes by hydroxycholesterol. Cholesterol is an important structural component of mammalian cells[100], while its oxygenated derivatives, including 24-, 25- and 27-hydroxycholesterol, exert important biological functions[101] such as the modulation of cell permeability[102] and signalling pathways[103]. As a result of pathological alterations in hydroxycholesterol abundance and/or localization, their roles extend into tumorigenesis and cancer development[95,104].

Here, we predict interactions involving hydroxycholesterol with 13 TRs, including important regulators of proliferation, differentiation, inflammation and metabolism: CEBPB, CNBP, DNMT1, HMGN1, ILF2, PAWR, PGR, RBBP7, RELA, STAT1, STAT6, TP53 and USF2 (Supplementary Data 5). U2F2 is a member of the sterol regulatory element-binding proteins (SREBPs)[105,106] that sense and respond to sterol levels, while several others have previously been linked to cholesterol homeostasis: CEBPB[107], CNBP[108], HMGN1[109], STAT1[110,111], and TP53[96] (see also the above example, section 2.4.2).

Sedoheptulose 7-phosphate. The metabolite sedoheptulose 7-phosphate (S7P), is an intermediate of the non-oxidative PPP that can fuel glycolysis (via glyceraldehyde 3-phosphate) and nucleotide synthesis (via ribose 5-phosphate). In our interaction map, we predict two interactions with S7P: (1) with STAT3, which has been linked to rewired energy metabolism in cancer[112,113], and (2) with UHRF1, a prominent regulator of DNA methylation and mediator of DNA damage repair[114,115].

Supplementary Note

Image analysis: cell number quantification from bright-field images of adherent cells

Cell concentration and viability information can be used for monitoring proliferation rates, optimizing growth conditions and normalizing cell data, like in our case metabolomics data. Here, we describe the procedure used for processing time-lapse bright-field microscopy images of adherent cells during time-course growth experiments. With the proposed protocol, we aim at a rapid quantification of cell numbers in images of live adherent cell cultures. The procedure has been optimized to scale with the number of images collected during long-term time lapse microscopy experiments, consisting of typically thousands of frames, and to generalize to cell types with radically different morphologies, without customization from the operator.

Currently, multiple platforms are routinely used for cell number quantification. Several methods are destructive and require enzymatic cell detachment using trypsin, adding increased cost to researchers and limiting flexibility as well as throughput. Identifying cells directly from microscopy images (cell segmentation) is an attractive alternative, and several image processing analysis tool are now routinely used for quantitative single-cell biology[116–120]. However, most methods use fluorescence microscopy, can be computationally demanding and poorly scale with the number of images. Here we propose a label-free and non-destructive approach based on bright-field microscopy imaging.

The detection and segmentation of adherent cells from bright-field microscopy images represents a challenging task. This is because (i) intensity of the background is often similar to the intensity of the cells, (ii) illumination is uneven and cells often have a surrounding bright white area (i.e. halo), (iii) contrast is poor and makes cell boundaries poorly defined, especially among sets of spatially close cells.

Here we acquired and processed bright-field images using a TECAN Spark 10M plate reader. For each well, 25 images were acquired and then stitched together, with a spatial resolution of 1.3 µm per pixel (panel a in Supplementary Figure 9). The subsequent steps of image analysis are described in detail below.

Our automatic procedure consists of 3 main steps:

(i) The image is preprocessed to improve contrast of cell borders (panels b-c in Supplementary Figure 9) using a series of filter masks in Matlab (e.g. imopen, imsharpen and imfilter).
(ii) Then we combined two techniques to perform cell segmentation. The first one is an empirically derived image gradient threshold selection method named Empirical Gradient Threshold (EGT) from[117]. Subsequently, we refined the segmentation using the Maximally Stable Extremal Regions (MSER) approach[121,122] using a previously developed code[123]. To speed up image acquisition by the plate reader, only the center of the well is used during the auto-focus procedure. Hence, image quality is higher in the central area, while images become more blurry (and hence difficult to segment) further away from the center of the well (panels a and d in Supplementary Figure 9**Error! Reference source not found.**). Therefore, to reduce the computational complexity and improve the quality of the segmentation, we

restricted the area of the image that is segmented to the central round area of each well (0.05 cm from the center of the well).

(iii) In the last step, we aim at estimating the characteristic size of adherent cells. Given the poor contrast of cell borders, segmentation of dense clusters of cells can fail to accurately separate individual cells. This often results in few, but large, unified blobs of cells. Other typical segmentation artefacts are associated to false identification of cells in the halo region. Such errors are more frequent, and typically associate with small segmented areas. While these wrongly segmented cells are typically a minor fraction with respect to correctly segmented cells, they can significantly bias the number of estimated cells. Hence, to cope with these classical segmentation errors, instead of directly counting segmented cells, we estimated the most characteristic cell size area based on the distribution of observed cell sizes, and use this estimate to compute total cell number as follows.

After cell segmentation, the distribution of cell sizes is computed by grouping cells into bins of equal size. The frequency associated to each bin is normalized by the maximum theoretical number of cells with the same size in the segmented well area. The most probable characteristic cell size area is then selected (red dashed line in panel e in Supplementary Figure 9). To account for the variability in cell sizes we performed different binning, adapted to the number of segmented cells. For each binning, we estimated the most frequent cell size, and calculated mean and standard deviation (Supplementary Figure 1e). The total cell number is then computed by dividing confluence (measured from the entire well) by the estimated characteristic cell size.

To test the performance and validity of our approach, we selected 24 images from different adherent cells types, at different confluency (images can be found at http://www.imsb.ethz.ch/research/zampieri-group/resources.html). For each image, cells were manually counted (panel f in Supplementary Figure 9), and compared to the number of cells predicted from our automatic image analysis method. The comparison from manually counted and automatically predicted cell numbers demonstrate excellent correlation (Pearson correlation = 0.95) (Supplementary Figure 1e), and an average coefficient of variance (CV = 16.1%) in line with traditionally accepted analysis methods[124].

While there are several freely available image analysis tools to perform cell segmentation analysis, most methods are optimized for phase contrast microscopy images[125], are computationally demanding[123] or necessitate parameter optimization based on the cell type imaged. The performance data presented here confirm that our methodology is capable of quantitatively estimating characteristic cell size and cell concentration across several adherent cell lines, without customization. Overall, our approach offers a rapid coarse-grained segmentation analysis, from which we can derive a rapid and accurate estimate of characteristic cell size area and cell number. It is worth noting that our image analysis tool can be easily adapted to other similar plate readers or similar microcopy setups. The code is implemented in Matlab and is available for download at http://www.imsb.ethz.ch/research/zampieri-group/resources.html.

Supplementary Figures

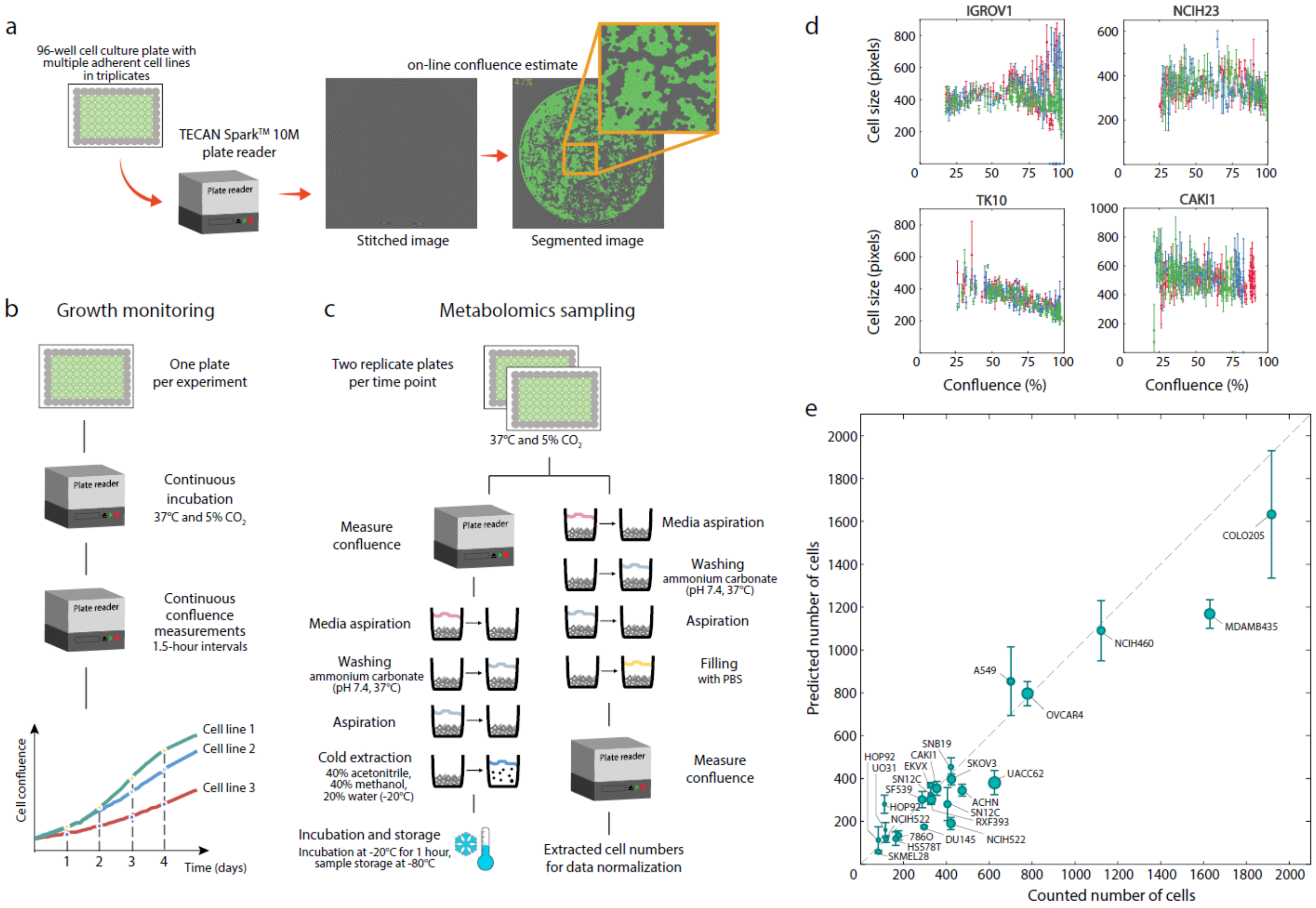

Supplementary Figure 1: Overview of the experimental workflow for high-throughput metabolome profiling in diverse adherent cell lines. The experimental workflow consists of a parallel extraction and cell number quantification of cell cultures grown in 96-well plates. **(a)** A TECAN Spark 10M plate reader is used to monitor cell growth by automatically acquiring bright-field images of a 96-well cell culture plate seeded with multiple adherent cell lines in triplicates. Confluence is estimated on-line by segmenting the percentage area of the well covered by cells (i.e. green area), using a manufacturer-proprietary algorithm, while cell numbers are estimated using an in-house software (see Supplementary Note and panels d-e). **(b)** Growth is continuously monitored on one replicate cell culture plate using time-lapse microscopy in a TECAN Spark 10M plate reader (37°C, 5% CO_2, see also panel a) to establish cell line- and condition-specific growth curves. **(c)** Sampling procedure. Two replicate culture plates are processed at each sampling time-point to collect cell extracts and to measure the extracted cell number for subsequent normalization of measured metabolite intensities. For metabolite extraction (left branch), the medium is aspirated from each well using a multi-channel aspirator, and the adherent cells are washed once with ammonium carbonate (37°C, 5% CO_2) to remove residual medium components. After aspiration, ice-cold extraction solvent (40:40:20 methanol:acetonitrile:water and 25 µM phenyl hydrazine, -20°C) is added to each well. The plate is immediately sealed, placed at -20°C for 1 hour, and subsequently stored at -80°C until analysis. Metabolome profiles are generated using a non-targeted mass spectrometry platform based on flow-injection time-of-flight mass spectrometry (FIA-TOFMS). The second plate (right branch) is processed identically, but is filled with warm phosphate-buffered saline (PBS, 37°C) and immediately subjected to cell confluence measurement to determine the number of cells extracted in each well. **(d)** Estimating adherent cell size from bright-field microscopy images acquired using a TECAN Spark 10M multi-well microplate reader. The algorithm is described in detail in the Supplementary

Note. The plot shows the dependence of cell size on cell confluence in three biological replicates (plotted in red, green and blue, respectively) of four representative cell lines from the NCI-60 panel of cancer cell lines. Cell size estimates in each time-lapse frame are plotted against cell confluence at the time of acquisition. Above 80% confluence, cells become difficult to segment, resulting in higher variation in cell sizes and a slight cell shrinkage. **(e)** Comparison of manually counted cell numbers with automatically determined cell counts derived by dividing the total area of the well covered by cells (i.e. well surface area multiplied by cell confluence in %) by the average adherent cell size (see panel d and Supplementary Note). Results are shown for twenty-four bright-field images from 21 different NCI-60 cell lines. The selected cell lines exhibit radically different morphologies, and images were taken at different levels of confluence. The images used for validation can be downloaded alongside with Matlab code from http://www.imsb.ethz.ch/research/zampieri-group/resources.html. It is worth noting that the detection and segmentation of adherent cells from bright-field microscopy images represents a challenging task. Most methodologies currently available are computationally time-consuming and require the user to optimize a set of unintuitive parameters to improve the segmentation of one specific cell line/condition. Our methodology aims at finding the best compromise to avoid any optimization of parameters and still achieve good performance in the estimates of cell numbers. The performance of our algorithm could be slightly improved if parameters were tuned on each cell line independently. However, this would require the user to perform a tedious and not always simple parameter tuning for each cell line/image, making such approach unsuitable for the analysis of a large number of images and cell lines. Here, we provide the community with a simple and intuitive tool to perform segmentation of large numbers of bright-field microscopy images of adherent cell lines without the need for customization.

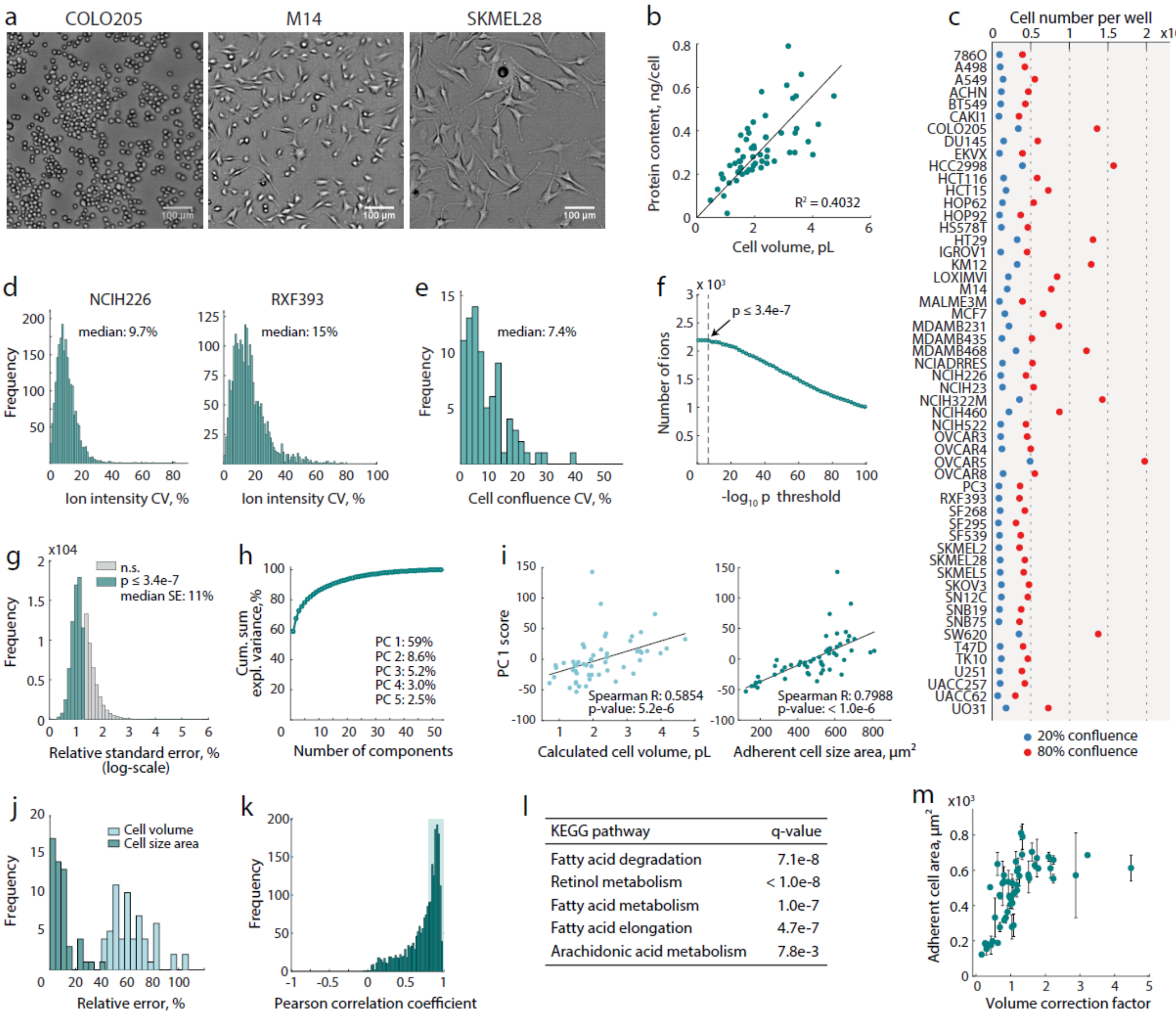

Supplementary Figure 2. Normalization of metabolomics data. (a) Bright-field microscopy images of adherent cultures of COLO205 colon, as well as M14 and SKMEL28 melanoma cells. These representative examples demonstrate the large differences in morphology and size that can be observed among cell lines from different and even similar tissue types. Images were acquired in a TECAN Spark 10M plate reader (37°C, 5% CO_2). **(b)** While there is a general dependency of protein content on the cell volume, cells with the same volume can exhibit up to 3-fold differences in protein content, making cellular protein content an inaccurate proxy of cell volume. **(c)** Minimum and maximum number of extracted cells in samples used for FIA-TOFMS analysis. Owing to the platform's high sensitivity, cell numbers ranging from as little as 10 000 cells to 200,000 cells per well can be used for metabolome profiling. Cell numbers were calculated from cell confluence divided by the characteristic adherent cell size. Characteristic areas covered on average by a single adherent cell were estimated for each cell line using the herein-presented image analysis tool (see Supplementary Note). The variation in extracted cell number between cell lines reflects considerable differences in cell sizes that are corrected during data normalization, as described in detail in the Methods section. **(d)** Variation in raw MS intensity data, shown for two representative cell lines from the NCI-60 panel of human tumor cell lines. Each plot shows the coefficients of variation (CV, in %) of 2181 ions per cell line, calculated across three replicate samples at the same time-point. Across all cell lines, the median variation of raw MS ion intensities is 13% (CV). The data shown represent raw MS intensities prior to any normalization, exhibiting the combined variance arising from differences in cell numbers (panel **c**) as well as fluctuations in MS signal

acquisition (instrumental noise). Of note, the degree of variance observed within each cell line overlaps with the observed variation in cell confluence between the same three biological replicates (panel **b**). Since we collected samples at different time points during growth, the number of extracted cells has a major contribution in MS-signal intensity and will play a crucial role in the subsequent normalization of raw MS data (see Figure 1 in the main text). **(e)** Variation in cell confluence across three biological replicates of 54 cell lines from the NCI-60 panel of human tumor cell lines. Coefficients of variation (CV, in %) were calculated across three biological replicate wells of the same cell line. The observed variance contains contributions from variations in initial cell seeding as well as in cell confluence measurements using bright-field microscopy imaging (using a TECAN Spark 10M plate reader) and image segmentation. The overall low degree of variation reflects the accuracy of cell confluence measurements. **(f)** Only ions with a significant (p-value ≤ 3.4e-7) linear dependency with cell number (function fitlm in Matlab) in more than 80% of cell lines were retained. **(g)** Distribution of relative standard errors of cell line-specific α-values, calculated from the standard error of the regression model (see Equation 2 in the main text). The data shown include α estimates for 2181 putatively annotated metabolites in cell lines where a significant dependency with the extracted cell number was detected (p ≤ 3.4e-7, see Figure 1b in the main text). A median CV of 11% demonstrates the robustness of this normalization approach. **(h-m)** Resolving cell volume differences in metabolomics data using Principal Component analysis (PCA). **(h)** PCA of the cell number-normalized dataset across 54 cell lines revealed a prominent pattern corresponding to almost 60% of total variance (PC1). **(j)** Correlation of PC1 scores with the cell volume calculated from previously published measurements of cell diameters when in suspension[126], and with the characteristic adherent cell size area that we determined from bright-field microscopy images. **(j)** Distribution of relative errors in cell volumes and adherent cell size area among 54 cell lines, showing that the directly measured adherent cell sizes have a smaller error than estimates of cell volumes. Cell volume errors were derived from error propagation of the measurement error in cell diameter as reported in [126], while errors in cell size area are derived directly from the distribution of segmented cells across images acquired between a confluence of 20 and 80% (see Supplementary Figure 1 and Supplementary Note). **(k)** Histogram of annotated metabolites correlating with PC1 scores. A subset of 987 metabolites that significantly correlate with PC1 (and consequently with cell size) are highlighted (i.e. Pearson correlation > 0.8 and p < 0.05) (see panel **a**). **(l)** KEGG pathway enrichment analysis revealed a strong over-representation of PC1-correlated metabolites in fatty acid metabolism. **(m)** Correlation of the calculated cell volume correction factors (based on 987 PC 1-associated annotated metabolites), with the adherent cell size area determined from bright-field image analysis. Error bars reflect the standard deviation of the adherent cell size area.

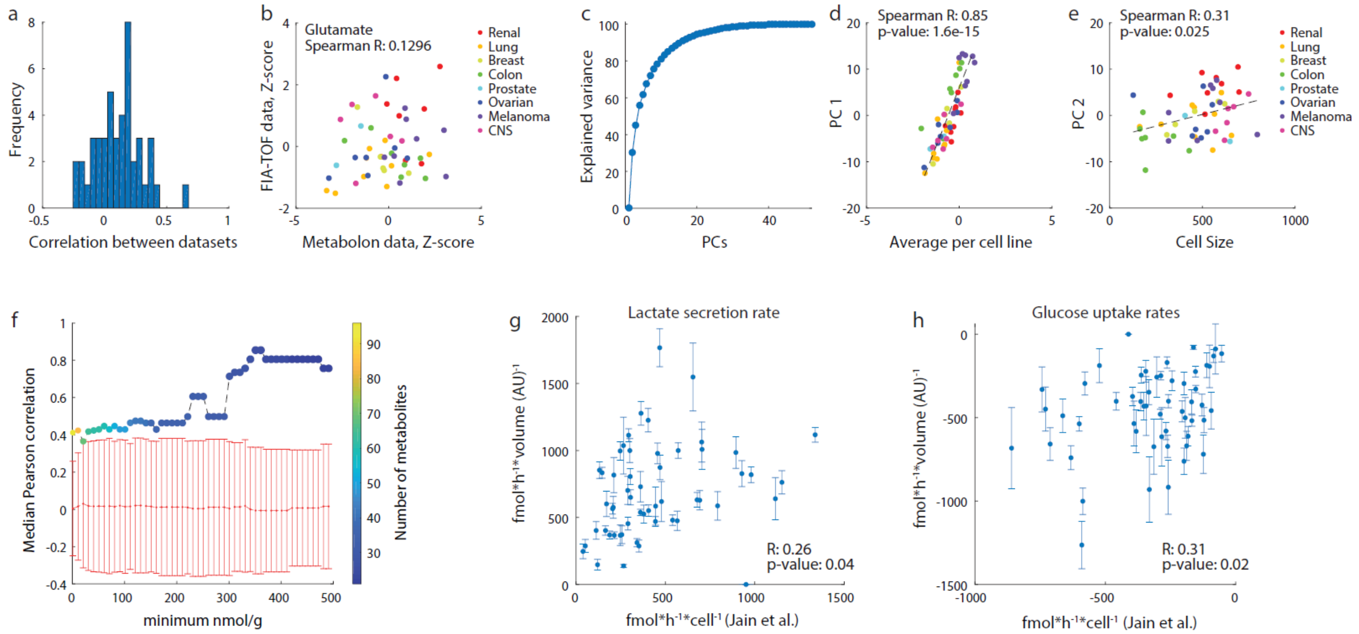

Supplementary Figure 3. Comparison of NCI-60 metabolome profiles with other metabolome resources. (a-e) Here we compare the metabolic profiles measured using our approach and previous metabolic profiles measured by the Metabolon company and available at the NCI DTP Molecular Target Program (https://wiki.nci.nih.gov/display/ncidtpdata/molecular+target+data). We detected 45 metabolites annotated to KEGG identifiers common between the Metabolon and our dataset. **(a)** Distribution of Spearman correlation between individual metabolite profiles across 53 NCI-60 cell lines. While most metabolites exhibit a positive correlation between the two datasets, overall we found a poor similarity between the metabolic profiles (Spearman correlation: 0.144 and p-value: 3.6e-12). **(b)** Glutamate relative metabolic profiles in the Metabolon and our dataset. Measurements from both datasets were used as Z-scores across 53 cell lines. While glutamate is one of the most abundant metabolite in the cell[8] and hence it represents a good term of comparison, it exhibits only poor correlation. A major difference between the two datasets is that we identify on average larger relative concentration of glutamate in central nervous systems cell lines (i.e. CNS), possibly reflecting the key role of glutamate as a neurotransmitter in the mammalian central nervous system. This observation is supported by independent data on mouse tissues (see panel f). **(c)** PCA analysis on the Metabolon dataset. We found two major Principal Components (PCs) that account for ~40% of the total variance. **(d)** First component (PC1) scores are plotted against the average metabolite levels for each cell line. This comparison shows that most metabolites exhibit similar patterns across cell lines. **(e)** The second component is plotted against cell size. The significant (p-value<0.05) correlation with cell size suggest that these data are not normalized to take into account differences in cell size. **(f)** Here we compare our metabolome dataset with a previously published metabolomics dataset[127] measuring the concentrations of 296 metabolites in 11 different tissue types of mouse: testis, pancreas, plasma, heart, kidney, liver, lung, spleen, thymus, cerebella, cerebra. Three tissue types: kidney, lung and cerebra, are in common with renal, lung and CNS tissues of origin in the NCI-60 cell lines. Since metabolite concentrations in [127] are given in absolute amounts per gram tissue, and not per cell volume, we used our data before cell volume correction (i.e. relative abundance per cell) for the comparison. To compare the relative values across tissues, we first take the mean values of metabolite intensities in NCI-60 cell lines of the same tissue types. Next, we calculate the median Pearson correlation for the 153 metabolites in common between the two datasets. Expected correlation values were estimated by randomly selecting mouse tissue types and average and standard deviation across 1000 permutations are reported (red line). To avoid artefacts related to sensitivity, we repeated this calculation for metabolites with an increasing minimum

absolute concentration (nmol/g) as measured in ref. [127]. We found that for metabolites with a concentration in at least one tissue type above 260 nmol/g, the median correlation with our data is above 0.5 and was larger than random. **(g)** Here we compare the results of our quantitative measurements of glucose uptake- and lactate secretion rates (y-axis) with measurements obtained for 53 cell lines in the work of Jain and colleagues[4] (x-axis). Two are the main differences between the two datasets: Firstly, instead of measuring only end-point concentrations of glucose and lactate in the spent medium, we measured changes in concentrations every 24 hours for approximately 5 days of cell growth. A linear model relating ion intensity to growth-normalized time (cell number divided by growth rate) were fitted, allowing for a more robust and quantitative assessment of uptake- and secretion rates. Secondly, instead of reporting the flux per cell, as in ref. 4, we correct for differences in cell volumes using the correction factor derived in our metabolomics approach (Supplementary Figure 2). Hence, we derive a relative measure of rates for both glucose uptake and lactate secretion expressed in fmol/h/volume (AU).

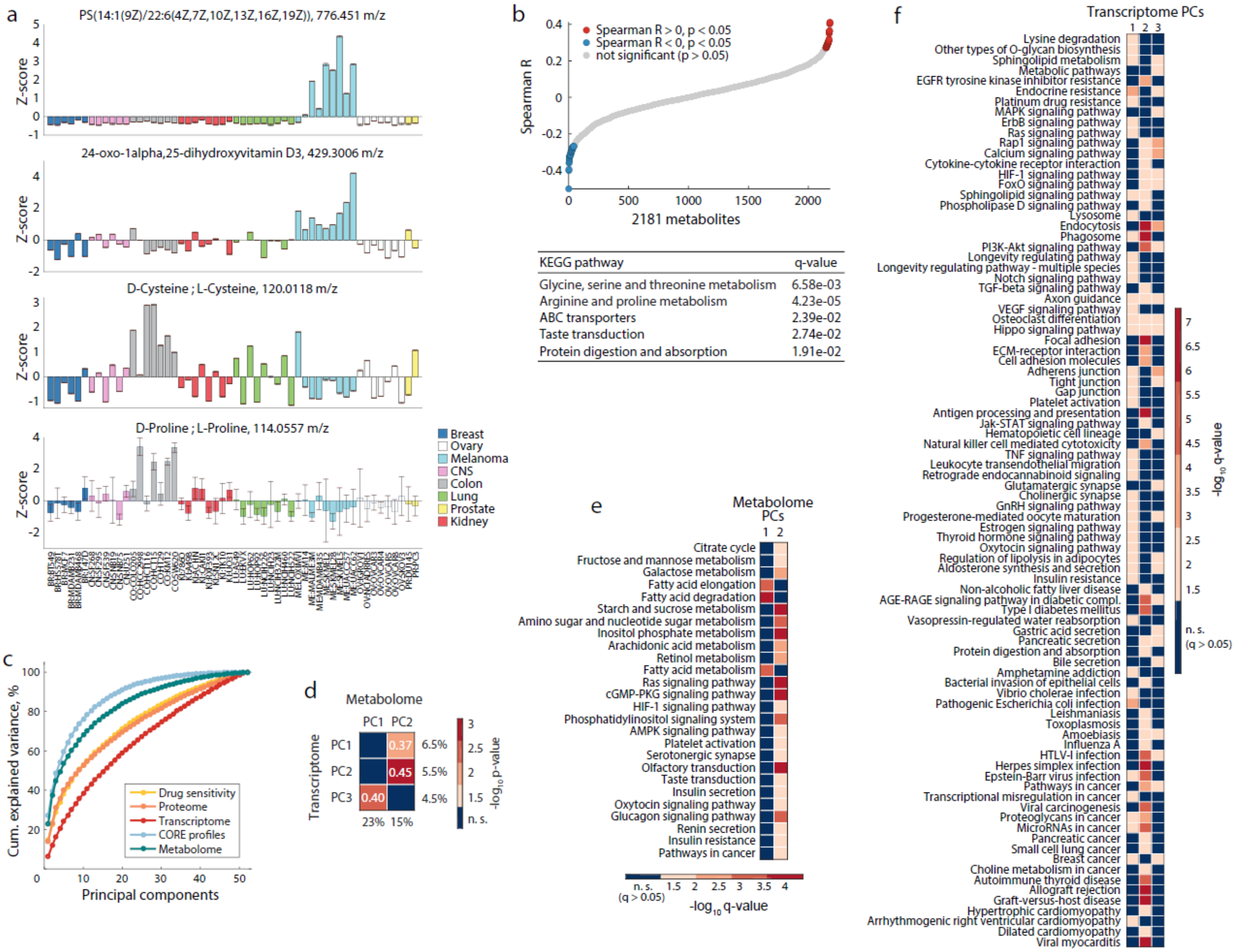

KEGG pathway	q-value
Glycine, serine and threonine metabolism	6.58e-03
Arginine and proline metabolism	4.23e-05
ABC transporters	2.39e-02
Taste transduction	2.74e-02
Protein digestion and absorption	1.91e-02

Supplementary Figure 4. Comparison of metabolic heterogeneity across cell lines and different layers of cell biological information. (a) Four examples for metabolites exhibiting tissue-dependent abundances (ANOVA q-value < 0.05, corrected for multiple tests). Relative metabolite abundances (y-axis) are given as Z-scores across the 54 cell lines, with error bars reflecting the standard deviation across 3 biological replicates. **(b)** Correlation of doubling times with relative metabolite abundances across the 54 adherent cell lines from the NCI-60 panel. Metabolites were ranked by the Spearman correlation coefficient (Supplementary Data 1). Metabolites that exhibit the largest correlation with growth rate across cell lines were tested for an overrepresentation in KEGG pathways, and were significantly enriched (q-value < 0.01) for intermediates in amino acids biosynthetic pathways (Supplementary Figure 3), possibly reflecting essential biochemical requirements for rapid cancer cell proliferation[4,128,129]. Only pathways with a q-value (corrected for multiple tests) below 0.05 are shown in the table. **(c)** Major trends in transcriptome, proteome, metabolome and drug sensitivity profiles and the corresponding fraction of explained variance were extracted using principal component analysis (PCA) of, as well as uptake/secretion profiles of 140 major substrates/by-products[4]. A high number of distinct trends (principal components) to explain most of the observed variance (e.g. 90%) in the data set indicates complex underlying patterns and high heterogeneity among cell lines. **(d)** The two main trends (principal components, PCs) in metabolic profiles across the 54 cell lines (≥ 10% explained variance each, see also panel **e**) correlate with major changes in gene expression levels (PCs 1, 2 and 3). Percentages represent the fraction of the total variance in the dataset explained by individual PCs. Spearman correlations were calculated between pairs of metabolome and transcriptome PCs (PC scores across cell lines). Box color corresponds to the correlation p-value, numbers in boxes show the absolute Spearman correlation coefficient for significant correlations (p-value < 0.05). n.s.: not significant (p-value > 0.05). **(e)** The strongest trend in metabolome profiles

(PC1, 23% variance explained) corresponds to differences in the abundance of several metabolites involved in fatty acid metabolism and degradation. The second-largest principal component (PC2, 15% explained variance) consists of metabolites in nucleotide metabolism and signaling pathways involved in cell proliferation and adaptation, such as Ras, HIF-1 and AMPK. **(f)** The three strongest trends in transcriptomic profiles are related to cell-cell interactions and cell adhesion (adherens- and tight junctions, Rap1 signaling, ErbB-, Hippo-, VEGF signaling), sphingolipid metabolism and several signal transduction pathways involved in cancer metabolism, such as HIF-1, PI3K-Akt, Ras and FoxO.

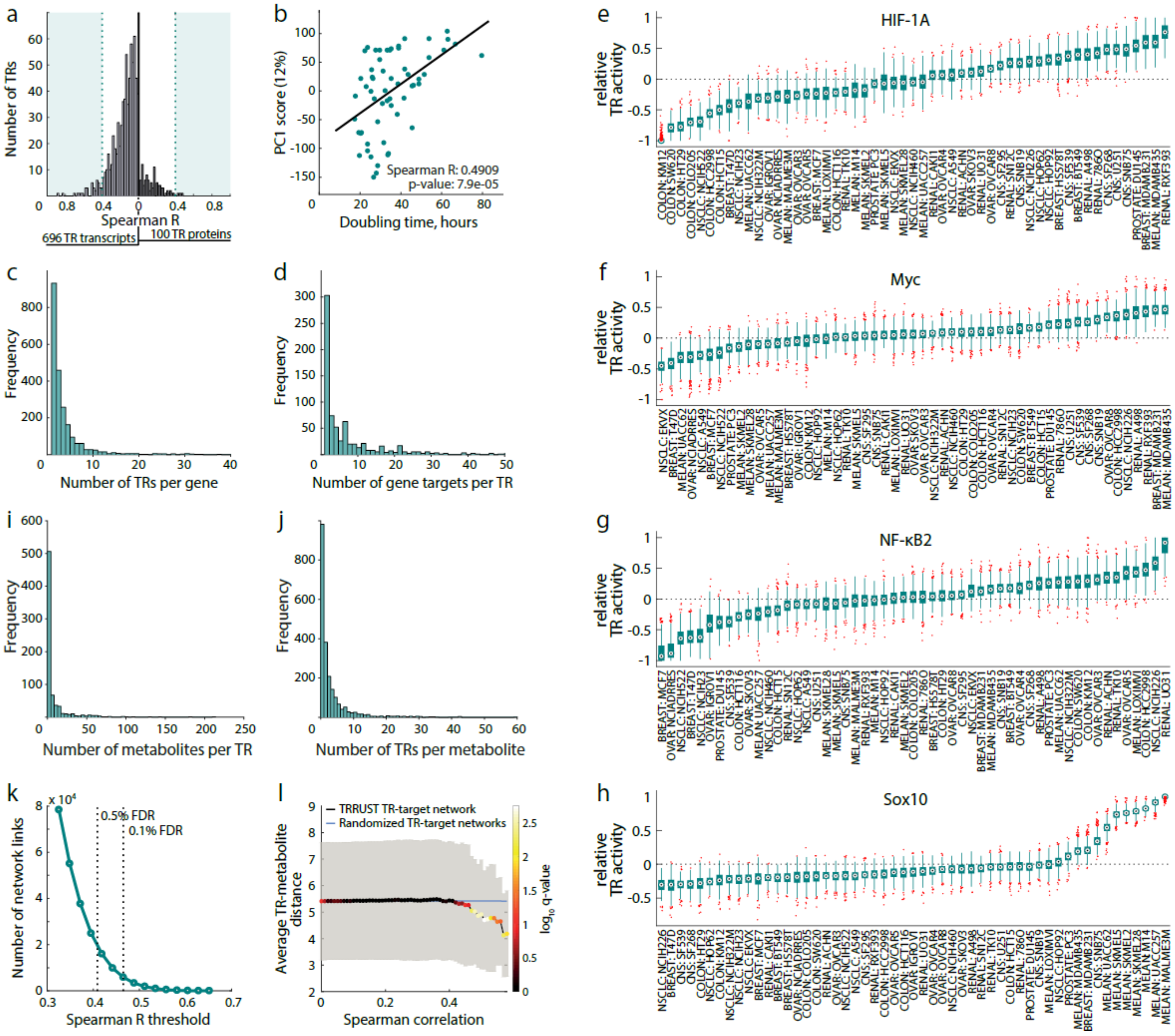

Supplementary Figure 5. Estimating TR activity *in silico* from target gene expression levels and known gene regulatory networks. (a) Correlation of calculated TR activity with gene expression levels (measured for 696 TRs) and protein abundance (measured for 100 TRs) across 53 adherent cell lines in the NCI-60 panel, showing that for the vast majority of TRs neither transcript nor protein abundance is an adequate predictor of TR activity. **(b)** The major trend in the transcript-omics data set used for NCA (12% explained variance) shows a strong correlation with cell line doubling time, reflecting a pleiotropic effect of growth rate on gene expression levels[130]. **(c-d)** Connectivity in the TR-gene network used in Network Component Analysis (NCA), including 728 TRs and 2209 unique target genes. Only 25 TRs (3.4%) have more than 50 gene targets, including Sp1, NF-κB, TP53, Jun and Myc. Each gene is on average controlled by three TRs (median: 2), while 13.7% of genes are connected to more than five TRs. **(e-h)** Example TR activity profiles across 53 cell lines calculated using NCA. Our implementation of NCA delivers median and standard deviation of a relative (i.e. within an unknown scaling factor) measure of TR activity between -1 and 1 (see Methods section for full detail). **(i-j)** Connectivity in the TR-metabolite association network (FDR ≤ 0.1%) generated by correlating metabolite abundances and calculated TR activity across 53 cell lines. The average degree (i.e. number of interaction partners) is 2.7 and 8.0 for metabolites (panel **i**) and transcriptional regulators (panel j), respectively. (k) Number of network links retained in the TR-metabolite association network at different false-discovery rates. Of note, no threshold was applied to the correlation-based network provided in Supplementary

Data 3. Upper ends of 99.5 and 99.9% confidence intervals were calculated using a bootstrapping approach. The cell lines in the metabolome data set were randomized by resampling 100 times with replacement, and Spearman correlation coefficients were calculated for each randomized data set. Correlation coefficients exceeding the maximum value among 99.5 and 99.9% (0.5 and 0.1% FDR, respectively) lowest correlation coefficients were obtained from the pooled list of absolute correlation coefficients. **(I)** Analysis of correlations between metabolite levels and TR activity profiles in relation to their distance between TR and enzyme targets in the stoichiometric network[131]. Only TR-target genes interactions listed in the TRRUST interaction database[132] were considered. The black line represents the average pairwise TR-metabolite distance (y-axis) at different levels of correlation between metabolite abundance and TR activity (x-axis). The blue line represents the average TR-metabolite distance in 10.000 randomized stoichiometric networks. Data points are color-coded to reflect the significance (q-value, corrected for multiple tests) of TR-metabolite proximity in the stoichiometric network as compared to the randomized networks.

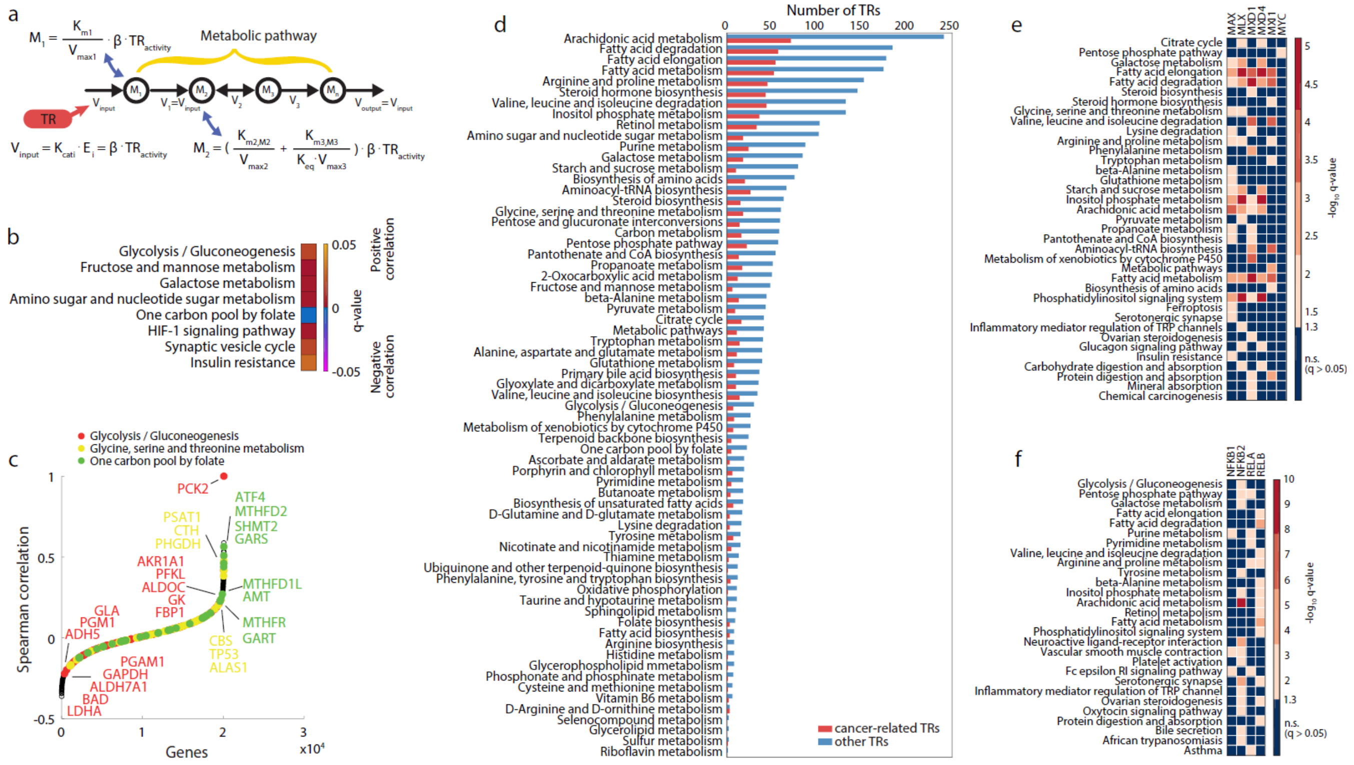

Supplementary Figure 6. Mapping TR activity to metabolic phenotypes. (a) Toy model in which a TR can directly modulate the flux (v) of a metabolic pathway. The pathway consists of 4 metabolic intermediates (M) and five enzymatic reactions governed by Michaelis-Menten kinetics. **(b)** Top 5% of metabolites whose abundance correlated with glycolytic flux (i.e. glucose uptake and lactate secretion, see main text Figure 2e) were tested for a significant (q-value, corrected for multiple tests) enrichment in KEGG metabolic pathways, separately for positively and negatively correlated metabolites (yellow to red and purple to blue color ranges, respectively). **(c)** Correlation of expression levels of SRCAP-regulated phosphoenolpyruvate carboxykinase 2 (PCK2) with more than 20.000 genes across cell lines in the CCLE. **(d)** Number of TRs whose associated metabolites show an over-representation in KEGG metabolic pathways. Only pathways with at least one associated TR are shown. The bar length corresponds to the number of TRs that show an overrepresentation (q-value < 0.05) of associated metabolites in the pathway. Cancer-related TRs (158 TRs) were defined based on the COSMIC cancer gene census[133]. Enrichment analysis highlights metabolic pathways where a significant number of metabolic intermediates exhibit coordinated changes the given TR's activity (see also main text Figure 3a). **(e-f)** Examples for TR-pathway associations: MYC and related proteins (panel e, the "MYC interactome"), and subunits of the NF-κB transcription factor complex (panel f) to KEGG metabolic pathways. These specific examples are further discussed in the Supplementary Discussion. Only pathways showing a significant overrepresentation (q-value < 0.05, corrected for multiple hypothesis testing) among metabolites associated with at least one of the TRs are shown in each panel. Colored boxes in shades of red reflect q-value significance, while blue squares correspond to non-significant links (no overrepresentation among associated metabolites, q-value > 0.05).

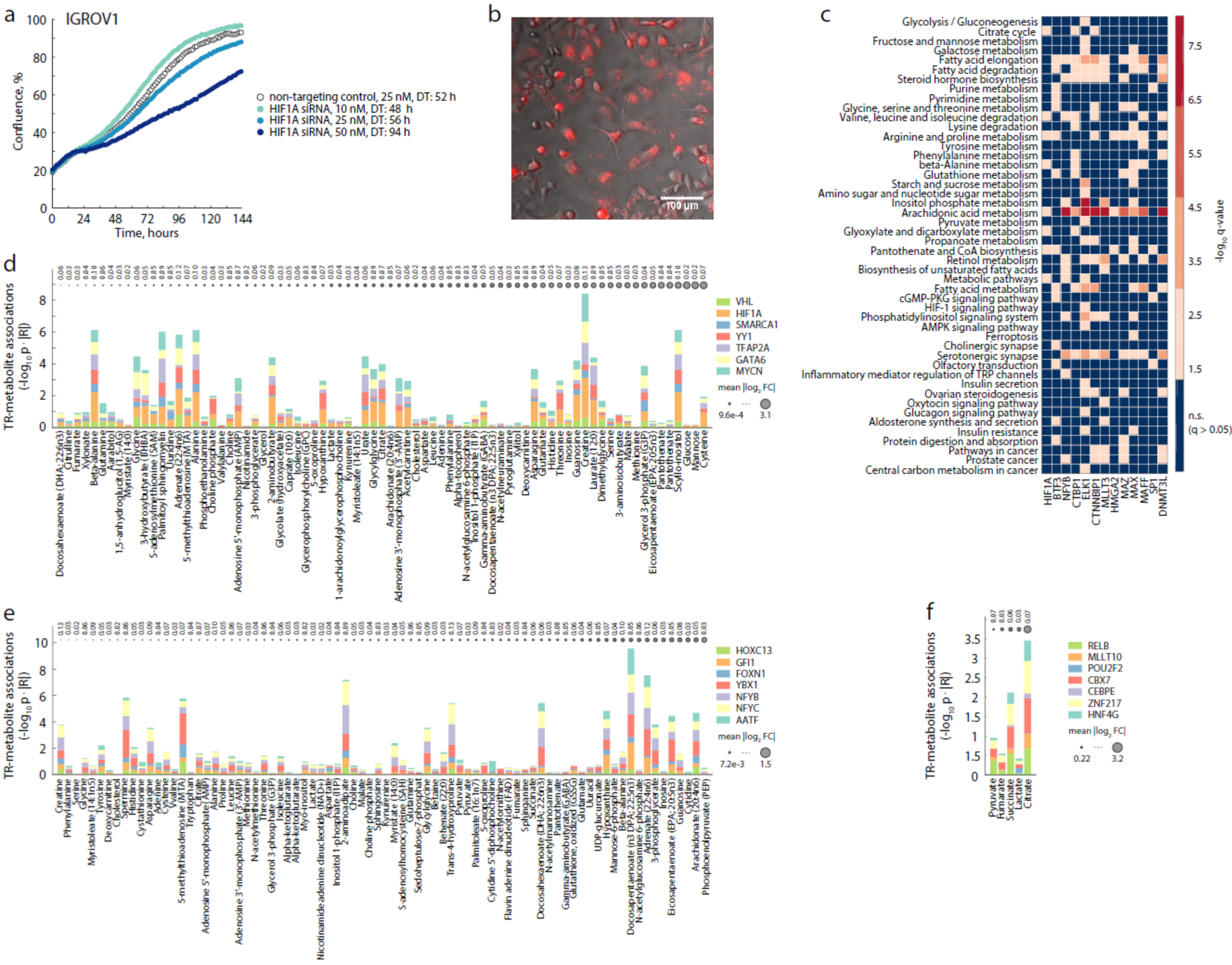

Supplementary Figure 7. Reverse-engineering of altered TR activity from metabolic profiles. (a) To test the ability of our TR-metabolite association network to resolve altered TR activity, we perturbed HIF-1A mRNA levels in IGROV1 ovarian cancer cells using RNAi. To that end, we transfected IGROV1 cells with three different siRNA concentrations (10, 25 and 50 nM). Control cultures were transfected with a non-targeting siRNA. Cell growth (i.e. cell confluence, y-axis) was monitored continuously using a TECAN Spark 10M plate reader for automated time-lapse microscopy (37°C, 5% CO_2). **(b)** Replicate IGROV1 cultures were transfected with a red fluorescent siRNA (see Supplementary Methods) to monitor cationic lipid-mediated transfection throughout the experiment using a Nikon Ti-E inverted fluorescence microscope. Cells were imaged directly in the 96-well plate without fixation. The image shows a representative section at 87 hours post-transfection in an overlay of a phase contrast and a fluorescence image. Red cells contain the fluorescent control siRNA, indicating successful transfection of IGROV1 cells (transfection efficiency: 92%). **(c)** Twelve TRs yielding a higher prediction score than HIF-1A share a strong association with fatty acid metabolism and may reflect effects of lipid transfection. The association to TCA cycle, however, is confined to HIF-1A and only two other TRs with a higher score than HIF-1A, CTBP1 and CTNNBIP1. **(d-f)** Inferring TR drivers of metabolic rearrangement observed in patient cohorts (matched tumor- and adjacent normal tissue pairs) from clear-cell renal cell carcinoma[49] (panel d, 138 patients), lung cancer[51] (panel e, 21 patients), and colon cancer[65] (panel f, 10 patients). A detailed description is provided in the Supplementary Discussion. The strength of TR-metabolite associations (product of correlation coefficient and $-\log_{10}$ p-value) is plotted for each quantified metabolite in the datasets (x-axis) for the seven most significant TR drivers identified (see Methods section and Figure 4 in the main text). Metabolites are sorted by the median absolute fold-change between tumor and adjacent normal tissue across

patients. The size of grey dots reflects the magnitude of the fold-change in log_2-scale. The numbers listed above the grey dots reflect the median association to the metabolite in our TR-metabolite association network, i.e. a small number indicates that the majority of TRs shows weak association to the metabolite, while the identified top-ranking TRs show stronger associations.

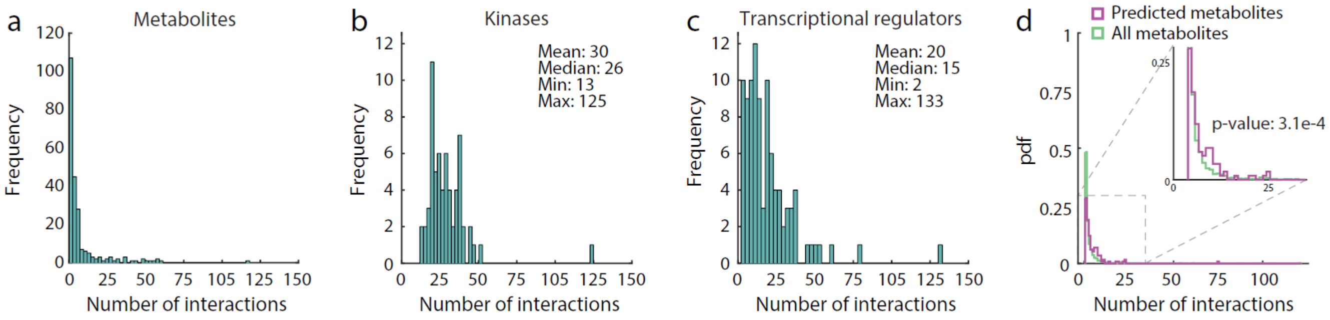

Supplementary Figure 8. A network of predicted interactions implying metabolites and kinases as potential effectors of TR activity. The different panels show the connectivity (number of interactions) of metabolites (panel **a**), kinases (panel **b**) and transcriptional regulators (panel **c**) in the predicted regulatory interaction network (Figure 4 in the main text). On average, each metabolite engaged in 8 interactions (median: 3), while kinases on the other hand were predicted to be more ubiquitous effectors (13-125 interactions per kinase). Network links were calculated based on non-linear model fitting analysis (see Methods section for full detail), where variance in TR activity across 53 cell lines was modeled as a function of the protein abundance of a given TR, and the additional activating or inhibiting action of a metabolite and/or a kinase, alone or in combinations. In total, we considered 100 TRs and 64 kinases/phosphatases for which protein abundance data was available, and 230 metabolites exhibiting largest variation across the 53 cell lines, resulting in 6,753,600 models. We tested for each model whether the interaction significantly improves the explained variance in TR activity across the 53 cell lines, and retained 1,888 network links (FDR ≤ 0.1%). **(d)** Probability density function (pdf) of the number of interactions reported for the metabolites in the predicted regulatory interaction network, as compared to all metabolites for which at least one allosteric interaction has been reported[9]. P-values report on the significance of the difference in interaction frequencies (Kolmogorov-Smirnov test) between the two groups of metabolites.

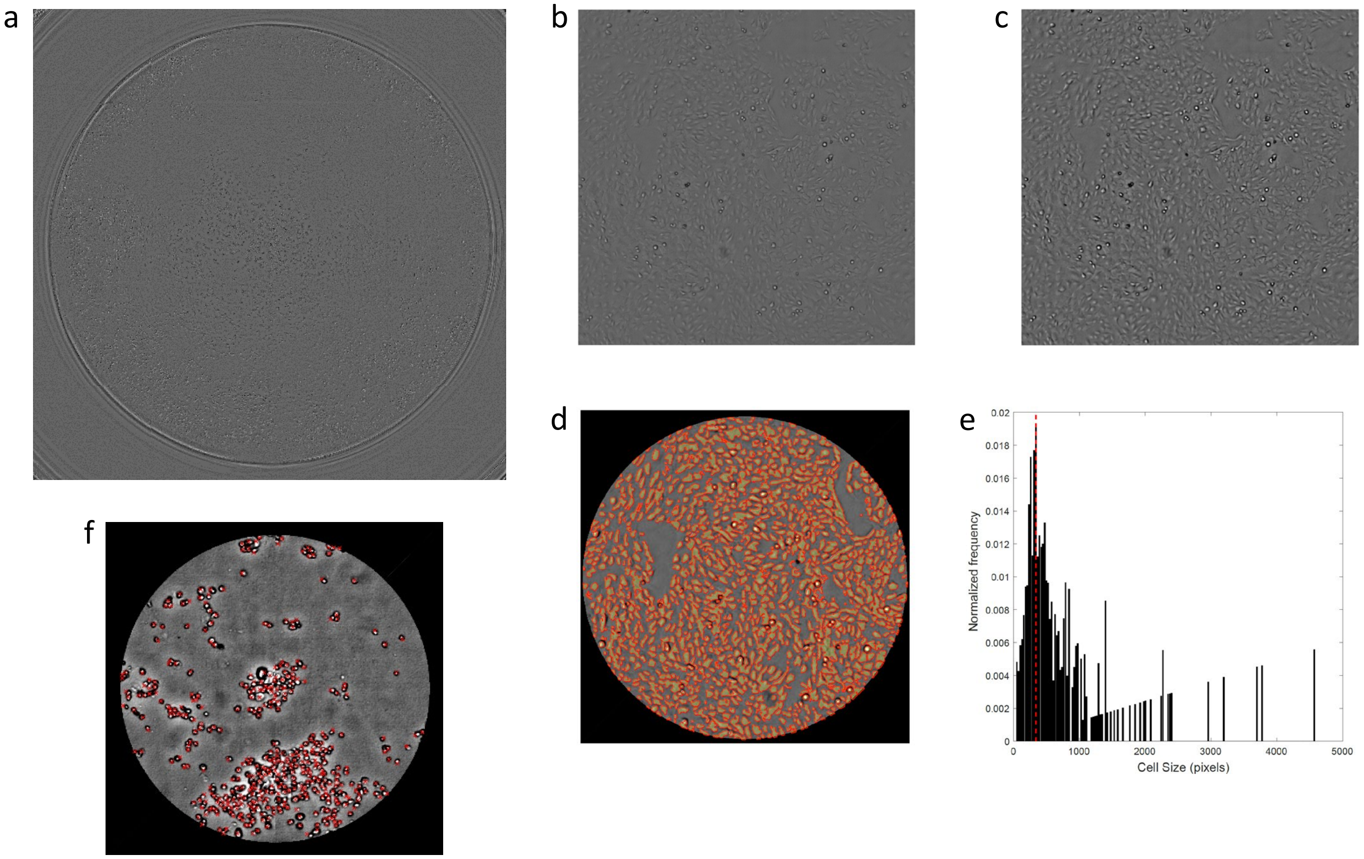

Supplementary Figure 9. Image analysis for cell number quantification from bright-field images of adherent cells. The software (Matlab code) is available for download at http://www.imsb.ethz.ch/research/zampieri-group/resources.html. **(a)** Example of raw bright field image of well bottom acquired with the TECAN Spark 10M plate reader. **(b-c)** Example of image sharpening. Raw image (panel b). Same image after the noise reduction and sharpening to improve the contrast and facilitate cell segmentation (panel c). **(d-e)** Example from segmentation analysis. Panel d shows the detected cells in green, and cell borders in red. In panel e, the histogram represents the normalized frequencies of cell sizes. The red dashed line indicates the cell size that is selected as the average cell size in this image. **(f)** Example from one of the image used for method validation. Manually counted cells are marked by a red cross.

Supplementary References

1. Pfister, T. D. *et al.* Topoisomerase I levels in the NCI-60 cancer cell line panel determined by validated ELISA and microarray analysis and correlation with indenoisoquinoline sensitivity. *Mol. Cancer Ther.* **8,** 1878–1884 (2009).

2. Guo, T. *et al.* Rapid proteotyping reveals cancer biology and drug response determinants in the NCI-60 cells. *bioRxiv* 268953 (2018). doi:10.1101/268953

3. Reinhold, W. C. *et al.* CellMiner: A Web-Based Suite of Genomic and Pharmacologic Tools to Explore Transcript and Drug Patterns in the NCI-60 Cell Line Set. *Cancer Res.* **72,** 3499–3511 (2012).

4. Jain, M. *et al.* Metabolite Profiling Identifies a Key Role for Glycine in Rapid Cancer Cell Proliferation. *Science* **336,** 1040–1044 (2012).

5. Storey, J. D. A Direct Approach to False Discovery Rates. *J. R. Stat. Soc. Ser. B* **64,** 479–498 (2002).

6. Dubuis, S., Ortmayr, K. & Zampieri, M. A framework for large-scale metabolome drug profiling links coenzyme A metabolism to the toxicity of anti-cancer drug dichloroacetate. *Commun. Biol.* **1,** 101 (2018).

7. Cascante, M. *et al.* Metabolic control analysis in drug discovery and disease. *Nat. Biotechnol.* **20,** 243–249 (2002).

8. Park, J. O. *et al.* Metabolite concentrations, fluxes and free energies imply efficient enzyme usage. *Nat. Chem. Biol.* **12,** 482–489 (2016).

9. Reznik, E. *et al.* Genome-Scale Architecture of Small Molecule Regulatory Networks and the Fundamental Trade-Off between Regulation and Enzymatic Activity. *Cell Rep.* **20,** 2666–2677 (2017).

10. Alam, M. T. *et al.* The self-inhibitory nature of metabolic networks and its alleviation through compartmentalization. *Nat. Commun.* **8,** ncomms16018 (2017).

11. Bieging, K. T., Mello, S. S. & Attardi, L. D. Unravelling mechanisms of p53-mediated tumour suppression. *Nat. Rev. Cancer* **14,** 359–370 (2014).

12. Levine, A. J. & Oren, M. The first 30 years of p53: growing ever more complex. *Nat. Rev. Cancer* **9,** 749–758 (2009).

13. Kruiswijk, F., Labuschagne, C. F. & Vousden, K. H. p53 in survival, death and metabolic health: a lifeguard with a licence to kill. *Nat. Rev. Mol. Cell Biol.* **16,** 393 (2015).

14. Polyak, K., Xia, Y., Zweier, J. L., Kinzler, K. W. & Vogelstein, B. A model for p53-induced apoptosis. *Nature* **389,** 300–305 (1997).

15. Liu, W., Hancock, C. N., Fischer, J. W., Harman, M. & Phang, J. M. Proline biosynthesis augments tumor cell growth and aerobic glycolysis: involvement of pyridine nucleotides. *Sci. Rep.* **5,** 17206 (2015).

16. Phang, J. M. The Proline Regulatory Axis and Cancer. *Front. Oncol.* **2,** (2012).

17. Siu, F., Bain, P. J., LeBlanc-Chaffin, R., Chen, H. & Kilberg, M. S. ATF4 Is a Mediator of the Nutrient-sensing Response Pathway That Activates the Human Asparagine Synthetase Gene. *J. Biol. Chem.* **277,** 24120–24127 (2002).

18. D'Aniello, C. *et al.* A novel autoregulatory loop between the Gcn2-Atf4 pathway and L-Proline metabolism controls stem cell identity. *Cell Death Differ.* **22,** 1094–1105 (2015).

19. Calogero, A. *et al.* The Early Growth Response Gene EGR-1 Behaves as a Suppressor Gene That Is Down-Regulated Independent of ARF/Mdm2 but not p53 Alterations in Fresh Human Gliomas. *Clin. Cancer Res.* **7,** 2788–2796 (2001).

20. Baron, V., Adamson, E. D., Calogero, A., Ragona, G. & Mercola, D. The transcription factor Egr1 is a direct regulator of multiple tumor suppressors including TGF*β*1, PTEN, p53, and fibronectin. *Cancer Gene Ther.* **13,** 115–124 (2006).

21. Unoki, M. & Nakamura, Y. EGR2 induces apoptosis in various cancer cell lines by direct transactivation of BNIP3L and BAK. *Oncogene* **22,** 2172–2185 (2003).

22. Oeckinghaus, A. & Ghosh, S. The NF-κB Family of Transcription Factors and Its Regulation. *Cold Spring Harb. Perspect. Biol.* **1,** a000034 (2009).

23. Hai, T. The ATF Transcription Factors in Cellular Adaptive Responses. in *Gene Expression and Regulation* (ed. Ma, J.) 329–340 (Springer New York, 2006). doi:10.1007/978-0-387-40049-5_20

24. Stine, Z. E., Walton, Z. E., Altman, B. J., Hsieh, A. L. & Dang, C. V. MYC, Metabolism, and Cancer. *Cancer Discov.* **5,** 1024–1039 (2015).

25. Meyer, N. & Penn, L. Z. Reflecting on 25 years with MYC. *Nat. Rev. Cancer* **8,** 976 (2008).

26. Dang, C. V. MYC, Metabolism, Cell Growth, and Tumorigenesis. *Cold Spring Harb. Perspect. Med.* **3,** a014217 (2013).

27. Amati, B. *et al.* Oncogenic activity of the c-Myc protein requires dimerization with Max. *Cell* **72,** 233–245 (1993).

28. Eilers, M. & Eisenman, R. N. Myc's broad reach. *Genes Dev.* **22,** 2755–2766 (2008).

29. Conacci-Sorrell, M., McFerrin, L. & Eisenman, R. N. An Overview of MYC and Its Interactome. *Cold Spring Harb. Perspect. Med.* **4,** a014357 (2014).

30. Walker, W., Zhou, Z.-Q., Ota, S., Wynshaw-Boris, A. & Hurlin, P. J. Mnt–Max to Myc–Max complex switching regulates cell cycle entry. *J Cell Biol* **169,** 405–413 (2005).

31. Mercurio, F. & Manning, A. M. NF-kappaB as a primary regulator of the stress response. *Oncogene* **18,** 6163–6171 (1999).

32. Xia, Y., Shen, S. & Verma, I. M. NF-κB, an Active Player in Human Cancers. *Cancer Immunol. Res.* **2,** 823–830 (2014).

33. Kawauchi, K., Araki, K., Tobiume, K. & Tanaka, N. p53 regulates glucose metabolism through an IKK-NF-κB pathway and inhibits cell transformation. *Nat. Cell Biol.* **10,** 611–618 (2008).

34. Tornatore, L., Thotakura, A. K., Bennett, J., Moretti, M. & Franzoso, G. The nuclear factor kappa B signaling pathway: integrating metabolism with inflammation. *Trends Cell Biol.* **22,** 557–566 (2012).

35. Wang, V. Y.-F. *et al.* The Transcriptional Specificity of NF-κB Dimers Is Coded within the κB DNA Response Elements. *Cell Rep.* **2,** 824–839 (2012).

36. Wong, D. *et al.* Extensive characterization of NF-κB binding uncovers non-canonical motifs and advances the interpretation of genetic functional traits. *Genome Biol.* **12,** R70 (2011).

37. Wulczyn, F. G., Naumann, M. & Scheidereit, C. Candidate proto-oncogene bcl-3 encodes a subunit-specific inhibitor of transcription factor NF-κB. *Nature* **358,** 597–599 (1992).

38. Harding, H. P. & Lazar, M. A. The orphan receptor Rev-ErbA alpha activates transcription via a novel response element. *Mol. Cell. Biol.* **13,** 3113–3121 (1993).

39. Burris, T. P. Nuclear Hormone Receptors for Heme: REV-ERBα and REV-ERBβ Are Ligand-Regulated Components of the Mammalian Clock. *Mol. Endocrinol.* **22,** 1509–1520 (2008).

40. Cho, H. *et al.* Regulation of circadian behaviour and metabolism by REV-ERB-α and REV-ERB-β. *Nature* **485,** 123 (2012).

41. Kourtidis, A. *et al.* An RNA Interference Screen Identifies Metabolic Regulators NR1D1 and PBP as Novel Survival Factors for Breast Cancer Cells with the ERBB2 Signature. *Cancer Res.* **70,** 1783–1792 (2010).

42. Mei, C. D. *et al.* Dual inhibition of REV-ERBβ and autophagy as a novel pharmacological approach to induce cytotoxicity in cancer cells. *Oncogene* **34,** 2597 (2015).

43. Kojetin, D. J. & Burris, T. P. REV-ERB and ROR nuclear receptors as drug targets. *Nat. Rev. Drug Discov.* **13,** 197 (2014).

44. Rini, B. I., Campbell, S. C. & Escudier, B. Renal cell carcinoma. *The Lancet* **373,** 1119–1132 (2009).

45. The Cancer Genome Atlas Research Network. Comprehensive molecular characterization of clear cell renal cell carcinoma. *Nature* **499,** 43–49 (2013).

46. Sato, Y. *et al.* Integrated molecular analysis of clear-cell renal cell carcinoma. *Nat. Genet.* **45,** 860 (2013).

47. Harlander, S. *et al.* Combined mutation in *Vhl*, *Trp53* and *Rb1* causes clear cell renal cell carcinoma in mice. *Nat. Med.* **23,** 869 (2017).

48. Linehan, W. M., Srinivasan, R. & Schmidt, L. S. The genetic basis of kidney cancer: a metabolic disease. *Nat. Rev. Urol.* **7,** 277 (2010).

49. Hakimi, A. A. *et al.* An Integrated Metabolic Atlas of Clear Cell Renal Cell Carcinoma. *Cancer Cell* **29,** 104–116 (2016).

50. The Cancer Genome Atlas Research Network. Comprehensive molecular profiling of lung adenocarcinoma. *Nature* **511,** 543–550 (2014).

51. Chaudhri, V. K. *et al.* Metabolic Alterations in Lung Cancer–Associated Fibroblasts Correlated with Increased Glycolytic Metabolism of the Tumor. *Mol. Cancer Res.* **11,** 579–592 (2013).

52. Du, P., Tang, F., Qiu, Y. & Dong, F. GFI1 Is Repressed by p53 and Inhibits DNA Damage-Induced Apoptosis. *PLOS ONE* **8,** e73542 (2013).

53. Khandanpour, C. *et al.* Growth Factor Independence 1 Antagonizes a p53-Induced DNA Damage Response Pathway in Lymphoblastic Leukemia. *Cancer Cell* **23,** 200–214 (2013).

54. Höpker, K. *et al.* AATF/Che-1 acts as a phosphorylation-dependent molecular modulator to repress p53-driven apoptosis. *EMBO J.* **31,** 3961–3975 (2012).

55. Gurtner, A., Manni, I. & Piaggio, G. NF-Y in cancer: Impact on cell transformation of a gene essential for proliferation. *Biochim. Biophys. Acta BBA - Gene Regul. Mech.* **1860,** 604–616 (2017).

56. Benatti, P. *et al.* A balance between NF-Y and p53 governs the pro- and anti-apoptotic transcriptional response. *Nucleic Acids Res.* **36,** 1415–1428 (2008).

57. Di Agostino, S. *et al.* Gain of function of mutant p53: The mutant p53/NF-Y protein complex reveals an aberrant transcriptional mechanism of cell cycle regulation. *Cancer Cell* **10,** 191–202 (2006).

58. Benatti, P. *et al.* NF-Y activates genes of metabolic pathways altered in cancer cells. *Oncotarget* **7,** 1633–1650 (2015).

59. Shibahara, K. *et al.* Nuclear Expression of the Y-Box Binding Protein, YB-1, as a Novel Marker of Disease Progression in Non-Small Cell Lung Cancer. *Clin. Cancer Res.* **7,** 3151–3155 (2001).

60. Kazanjian, A. *et al.* Growth Factor Independence-1 Is Expressed in Primary Human Neuroendocrine Lung Carcinomas and Mediates the Differentiation of Murine Pulmonary Neuroendocrine Cells. *Cancer Res.* **64,** 6874–6882 (2004).

61. Bhatlekar, S., Fields, J. Z. & Boman, B. M. HOX genes and their role in the development of human cancers. *J. Mol. Med.* **92,** 811–823 (2014).

62. The Cancer Genome Atlas Research Network. Comprehensive molecular characterization of human colon and rectal cancer. *Nature* **487,** 330–337 (2012).

63. Dienstmann, R. *et al.* Consensus molecular subtypes and the evolution of precision medicine in colorectal cancer. *Nat. Rev. Cancer* **17,** 79 (2017).

64. Pino, M. S. & Chung, D. C. The Chromosomal Instability Pathway in Colon Cancer. *Gastroenterology* **138,** 2059–2072 (2010).

65. Hu, J. *et al.* Heterogeneity of tumor-induced gene expression changes in the human metabolic network. *Nat. Biotechnol.* **31,** 522 (2013).

66. Karin, M. Nuclear factor-κB in cancer development and progression. *Nature* **441,** 431–436 (2006).

67. Cooks, T. *et al.* Mutant p53 Prolongs NF-κB Activation and Promotes Chronic Inflammation and Inflammation-Associated Colorectal Cancer. *Cancer Cell* **23,** 634–646 (2013).

68. Viennois, E., Chen, F. & Merlin, D. NF-κB pathway in colitis-associated cancers. *Transl. Gastrointest. Cancer* **2,** 21–29 (2012).

69. Cohen, P. A. *et al.* The dark side of ZNF217, a key regulator of tumorigenesis with powerful biomarker value. *Oncotarget* **6,** 41566–41581 (2015).

70. Pallante, P. *et al.* The loss of the CBX7 gene expression represents an adverse prognostic marker for survival of colon carcinoma patients. *Eur. J. Cancer* **46,** 2304–2313 (2010).

71. Bao, Z. *et al.* CBX7 negatively regulates migration and invasion in glioma via Wnt/β-catenin pathway inactivation. *Oncotarget* **8,** 39048–39063 (2017).

72. Mahmoudi, T. *et al.* The Leukemia-Associated Mllt10/Af10-Dot1l Are Tcf4/β-Catenin Coactivators Essential for Intestinal Homeostasis. *PLOS Biol.* **8,** e1000539 (2010).

73. Lee, D.-F., Walsh, M. J. & Aguiló, F. ZNF217/ZFP217 Meets Chromatin and RNA. *Trends Biochem. Sci.* **41,** 986–988 (2016).

74. Fleming, N. I. *et al.* SMAD2, SMAD3 and SMAD4 Mutations in Colorectal Cancer. *Cancer Res.* **73,** 725–735 (2013).

75. Glunde, K., Bhujwalla, Z. M. & Ronen, S. M. Choline metabolism in malignant transformation. *Nat. Rev. Cancer* **11,** 835 (2011).

76. Dever, T. E. Translation initiation: adept at adapting. *Trends Biochem. Sci.* **24,** 398–403 (1999).

77. Williams, B. R. PKR; a sentinel kinase for cellular stress. *Oncogene* **18,** 6112 (1999).

78. Li, G., Scull, C., Ozcan, L. & Tabas, I. NADPH oxidase links endoplasmic reticulum stress, oxidative stress, and PKR activation to induce apoptosis. *J. Cell Biol.* **191,** 1113–1125 (2010).

79. Tong, L., Heim, R. A. & Wu, S. Nitric oxide: A regulator of eukaryotic initiation factor 2 kinases. *Free Radic. Biol. Med.* **50,** 1717–1725 (2011).

80. Bonnet, M. C., Weil, R., Dam, E., Hovanessian, A. G. & Meurs, E. F. PKR Stimulates NF-κB Irrespective of Its Kinase Function by Interacting with the IκB Kinase Complex. *Mol. Cell. Biol.* **20,** 4532–4542 (2000).

81. Zamanian-Daryoush, M., Mogensen, T. H., DiDonato, J. A. & Williams, B. R. G. NF-κB Activation by Double-Stranded-RNA-Activated Protein Kinase (PKR) Is Mediated through NF-κB-Inducing Kinase and IκB Kinase. *Mol. Cell. Biol.* **20,** 1278–1290 (2000).

82. Xie, M. *et al.* PKM2-dependent glycolysis promotes NLRP3 and AIM2 inflammasome activation. *Nat. Commun.* **7,** 13280 (2016).

83. Strano, S. *et al.* Mutant p53: an oncogenic transcription factor. *Oncogene* **26,** 2212 (2007).

84. Fridman, J. S. & Lowe, S. W. Control of apoptosis by p53. *Oncogene* **22,** 9030 (2003).

85. Reinhardt, H. C. & Schumacher, B. The p53 network: cellular and systemic DNA damage responses in aging and cancer. *Trends Genet.* **28,** 128–136 (2012).

86. Meek, D. W. Tumour suppression by p53: a role for the DNA damage response? *Nat. Rev. Cancer* **9,** 714 (2009).

87. Gottlieb, E. & Vousden, K. H. p53 Regulation of Metabolic Pathways. *Cold Spring Harb. Perspect. Biol.* **2,** a001040 (2010).

88. Vousden, K. H. & Ryan, K. M. p53 and metabolism. *Nat. Rev. Cancer* **9,** 691 (2009).

89. Okorokov, A. L. & Milner, J. An ATP/ADP-Dependent Molecular Switch Regulates the Stability of p53-DNA Complexes. *Mol. Cell. Biol.* **19,** 7501–7510 (1999).

90. Yoo, T., Lee, J., Chun, H. & Chi, S. α-lipoic acid prevents p53 degradation in colon cancer cells by blocking Nf-κb induction of Rps6ka4. *Anticancer. Drugs* **24,** 555–565 (2013).

91. Zeng, L. *et al.* Saturated Fatty Acids Modulate Cell Response to DNA Damage: Implication for Their Role in Tumorigenesis. *PLOS ONE* **3,** e2329 (2008).

92. Mandal, S., Mandal, A., Johansson, H. E., Orjalo, A. V. & Park, M. H. Depletion of cellular polyamines, spermidine and spermine, causes a total arrest in translation and growth in mammalian cells. *Proc. Natl. Acad. Sci.* **110,** 2169–2174 (2013).

93. Bhattacharya, S., Ray, R. M. & Johnson, L. R. Role of polyamines in p53-dependent apoptosis of intestinal epithelial cells. *Cell. Signal.* **21,** 509–522 (2009).

94. Ou, Y., Wang, S.-J., Li, D., Chu, B. & Gu, W. Activation of SAT1 engages polyamine metabolism with p53-mediated ferroptotic responses. *Proc. Natl. Acad. Sci.* **113,** E6806–E6812 (2016).

95. Kloudova, A., Guengerich, F. P. & Soucek, P. The Role of Oxysterols in Human Cancer. *Trends Endocrinol. Metab.* **28,** 485–496 (2017).

96. Raza, S. *et al.* The cholesterol metabolite 27-hydroxycholesterol regulates p53 activity and increases cell proliferation via MDM2 in breast cancer cells. *Mol. Cell. Biochem.* **410,** 187–195 (2015).

97. Soussi, T. & Wiman, K. G. TP53: an oncogene in disguise. *Cell Death Differ.* **22,** 1239 (2015).

98. Agarwal, M. K., Agarwal, M. L., Athar, M. & Gupta, S. Tocotrienol-Rich Fraction of Palm Oil Activates p53, Modulates Bax/Bcl2 Ratio and Induces Apoptosis Independent of Cell Cycle Association. *Cell Cycle* **3,** 200–199 (2004).

99. Ford, J. H. Saturated fatty acid metabolism is key link between cell division, cancer, and senescence in cellular and whole organism aging. *Age* **32,** 231–237 (2010).

100. Ikonen, E. Cellular cholesterol trafficking and compartmentalization. *Nat. Rev. Mol. Cell Biol.* **9,** 125 (2008).

101. Kulig, W., Cwiklik, L., Jurkiewicz, P., Rog, T. & Vattulainen, I. Cholesterol oxidation products and their biological importance. *Chem. Phys. Lipids* **199,** 144–160 (2016).

102. Kulig, W. *et al.* Cholesterol under oxidative stress—How lipid membranes sense oxidation as cholesterol is being replaced by oxysterols. *Free Radic. Biol. Med.* **84,** 30–41 (2015).

103. Leonarduzzi, G. *et al.* S3-2 - Modulation of cell signaling pathways by oxysterols in age-related human diseases. *Free Radic. Biol. Med.* **75,** S5 (2014).

104. Poli, G. & Biasi, F. Potential modulation of cancer progression by oxysterols. *Mol. Aspects Med.* **49,** 47–48 (2016).

105. Osborne, T. F. Sterol Regulatory Element-binding Proteins (SREBPs): Key Regulators of Nutritional Homeostasis and Insulin Action. *J. Biol. Chem.* **275,** 32379–32382 (2000).

106. Athanikar, J. N. & Osborne, T. F. Specificity in cholesterol regulation of gene expression by coevolution of sterol regulatory DNA element and its binding protein. *Proc. Natl. Acad. Sci.* **95,** 4935–4940 (1998).

107. Millward, C. A. *et al.* Mice With a Deletion in the Gene for CCAAT/Enhancer-Binding Protein β Are Protected Against Diet-Induced Obesity. *Diabetes* **56,** 161–167 (2007).

108. Rajavashisth, T. B., Taylor, A. K., Andalibi, A., Svenson, K. L. & Lusis, A. J. Identification of a zinc finger protein that binds to the sterol regulatory element. *Science* **245,** 640–643 (1989).

109. Postnikov, Y. V., Furusawa, T., Haines, D. C., Factor, V. M. & Bustin, M. Loss of the Nucleosome-Binding Protein HMGN1 Affects the Rate of N-Nitrosodiethylamine-Induced Hepatocarcinogenesis in Mice. *Mol. Cancer Res.* **12,** 82–90 (2014).

110. Matsumiya, T. & Imaizumi, T. How are STAT1 and cholesterol metabolism associated in antiviral responses? *JAK-STAT* **2,** e24189 (2013).

111. Blanc, M. *et al.* The Transcription Factor STAT-1 Couples Macrophage Synthesis of 25-Hydroxycholesterol to the Interferon Antiviral Response. *Immunity* **38,** 106–118 (2013).

112. Avalle, L., Camporeale, A., Camperi, A. & Poli, V. STAT3 in cancer: A double edged sword. *Cytokine* **98,** 42–50 (2017).

113. Yu, H., Lee, H., Herrmann, A., Buettner, R. & Jove, R. Revisiting STAT3 signalling in cancer: new and unexpected biological functions. *Nat. Rev. Cancer* **14,** 736 (2014).

114. Tian, Y. *et al.* UHRF1 Contributes to DNA Damage Repair as a Lesion Recognition Factor and Nuclease Scaffold. *Cell Rep.* **10,** 1957–1966 (2015).

115. Zhang, H. *et al.* A cell cycle-dependent BRCA1–UHRF1 cascade regulates DNA double-strand break repair pathway choice. *Nat. Commun.* **7,** 10201 (2016).

116. Bradbury, L. & Wan, J. W. L. A spectral k-means approach to bright-field cell image segmentation. in *2010 Annual International Conference of the IEEE Engineering in Medicine and Biology* 4748–4751 (2010). doi:10.1109/IEMBS.2010.5626380

117. Chalfoun, J. *et al.* Empirical gradient threshold technique for automated segmentation across image modalities and cell lines. *J. Microsc.* **260,** 86–99 (2015).

118. Dimopoulos, S., Mayer, C. E., Rudolf, F. & Stelling, J. Accurate cell segmentation in microscopy images using membrane patterns. *Bioinformatics* btu302 (2014). doi:10.1093/bioinformatics/btu302

119. Eliceiri, K. W. *et al.* Biological imaging software tools. *Nat. Methods* **9,** 697–710 (2012).

120. Meijering, E. Cell Segmentation: 50 Years Down the Road [Life Sciences]. *IEEE Signal Process. Mag.* **29,** 140–145 (2012).

121. Nistér, D. & Stewénius, H. Linear Time Maximally Stable Extremal Regions. in *Proceedings of the 10th European Conference on Computer Vision: Part II* 183–196 (Springer-Verlag, 2008). doi:10.1007/978-3-540-88688-4_14

122. Matas, J., Chum, O., Urban, M. & Pajdla, T. Robust wide-baseline stereo from maximally stable extremal regions. *Image Vis. Comput.* **22,** 761–767 (2004).

123. Buggenthin, F. *et al.* An automatic method for robust and fast cell detection in bright field images from high-throughput microscopy. *BMC Bioinformatics* **14,** 297 (2013).

124. BioTechniques - Precise and Accurate Counts and Viability Measurements Across Multiple Cell Lines Using the Muse™ Cell Count & Viability Assay. Available at: http://www.biotechniques.com/BiotechniquesJournal/2012/March/Precise-and-Accurate-Counts-and-Viability-Measurements-Across-Multiple-Cell-Lines-Using-the-Muse-Cell-Count-and-Viability-Assay/biotechniques-327917.html. (Accessed: 15th December 2016)

125. Su, H., Yin, Z., Huh, S. & Kanade, T. Cell segmentation in phase contrast microscopy images via semi-supervised classification over optics-related features. *Med. Image Anal.* **17,** 746–765 (2013).

126. Dolfi, S. C. *et al.* The metabolic demands of cancer cells are coupled to their size and protein synthesis rates. *Cancer Metab.* **1,** 20 (2013).

127. Sugimoto, M. *et al.* MMMDB: Mouse Multiple Tissue Metabolome Database. *Nucleic Acids Res.* **40,** D809–D814 (2012).

128. Hosios, A. M. *et al.* Amino Acids Rather than Glucose Account for the Majority of Cell Mass in Proliferating Mammalian Cells. *Dev. Cell* **36,** 540–549 (2016).

129. Pavlova, N. N. *et al.* As Extracellular Glutamine Levels Decline, Asparagine Becomes an Essential Amino Acid. *Cell Metab.* **27,** 428–438.e5 (2018).

130. Klumpp, S., Zhang, Z. & Hwa, T. Growth Rate-Dependent Global Effects on Gene Expression in Bacteria. *Cell* **139,** 1366–1375 (2009).

131. Thiele, I. *et al.* A community-driven global reconstruction of human metabolism. *Nat. Biotechnol.* **31,** 419–425 (2013).

132. Han, H. *et al.* TRRUST: a reference database of human transcriptional regulatory interactions. *Sci. Rep.* **5,** srep11432 (2015).

133. Forbes, S. A. *et al.* COSMIC: somatic cancer genetics at high-resolution. *Nucleic Acids Res.* **45,** D777–D783 (2017).
