# Supplementary material for: Metabolic profiling of cancer cells reveals genome-wide crosstalk between transcriptional regulators and metabolism
Source: Nat Commun. 2019 Apr 23;10:1841. doi: 10.1038/s41467-019-09695-9 (PMC6478870; doi:10.1038/s41467-019-09695-9)
Supplement: Supplementary file 8 — Description of Additional Supplementary Files [file 41467_2019_9695_MOESM8_ESM.docx]

**Title:** Supplementary Data 1.
**Description:** Metabolomics data for 54 adherent cell lines from the NCI-60 cancer cell line panel: relative intracellular abundances and glucose uptake- and lactate secretion rates.

**Title:** Supplementary Data 2.
**Description:** Relative TR activity profiles for 728 TRs in 53 NCI-60 cell lines.

**Title:** Supplementary Data 3.
**Description:** TR-metabolite association network, mapping to metabolic phenotypes and interpretation of in vivo metabolic reprogramming in cancer.

**Title:** Supplementary Data 4.
**Description:** Associations between TR activity and the sensitivity to FDA-approved drugs.

**Title:** Supplementary Data 5.
**Description:** Interactions involving metabolites and kinases that can modulate TR activity.

**Title:** Supplementary Data 6.
**Description:** Augmented TR-target network, derived from correlation of enzyme abundance with TR activity across 1037 cell lines in the Cancer Cell Line Encyclopedia (CCLE)
